# Antibody–drug conjugates in cancer therapy: current landscape, challenges, and future directions

**DOI:** 10.1186/s12943-025-02489-2

**Published:** 2025-11-03

**Authors:** Bonan Chen, Xiaohong Zheng, Jialin Wu, Guoming Chen, Jun Yu, Yi Xu, William K. K. Wu, Gary M. K. Tse, Ka Fai To, Wei Kang

**Affiliations:** 1 Department of Anatomical and Cellular Pathology, State Key Laboratory of Translational Oncology, Sir Y.K. Pao Cancer Center, The Chinese University of Hong Kong, Hong Kong SAR, China; 2 Institute of Digestive Disease, State Key Laboratory of Digestive Disease, Li Ka Shing Institute of Health Science, The Chinese University of Hong Kong, Hong Kong SAR, China; 3 CUHK-Shenzhen Research Institute, Shenzhen, China; 4 The Fourth Clinical Medical College of Guangzhou University of Chinese Medicine, Shenzhen, China; 5 School of Chinese Medicine, Li Ka Shing Faculty of Medicine, The University of Hong Kong, Hong Kong, China; 6 Department of Medicine and Therapeutics, The Chinese University of Hong Kong, Hong Kong SAR, China; 7 Department of General Surgery, The Second Affiliated Hospital of Harbin Medical University, Harbin, China; 8 Department of Anaesthesia and Intensive Care, The Chinese University of Hong Kong, Hong Kong, China

**Keywords:** Antibody-drug conjugate, Targeted cancer therapy, Payload design, Nanotechnology, Drug resistance

## Abstract

Antibody-drug conjugates (ADCs) have emerged as a transformative modality in oncology by combining the target specificity of antibodies with the high potency of diverse cytotoxic payloads. This review provides an integrative overview of ADCs, spanning from molecular design to clinical translation. We dissect the structural components, antibodies, linkers, and payloads, and elucidate their impact on pharmacokinetics, tumor selectivity, and therapeutic index. Mechanistic pathways, including antigen recognition, receptor-mediated internalization, payload release, and immunogenic cell death (ICD), are highlighted to provide context for ADC function. Clinically, ADCs have demonstrated efficacy across hematologic and solid malignancies, with 15 Food and Drug Administration (FDA) approvals and an expanding investigational pipeline. However, challenges persist, including antigen heterogeneity, resistance mechanisms, systemic toxicities, and manufacturing complexities. Emerging innovations such as bispecific ADCs, immune-stimulatory payloads, AI-guided design, and nanotechnology-enhanced delivery are reshaping the ADC landscape. Finally, we emphasize the necessity of diagnostic precision and rational combination strategies, while highlighting emerging innovations that collectively shape the future direction of next-generation ADC therapeutics.

## Introduction

**Fig.1:**
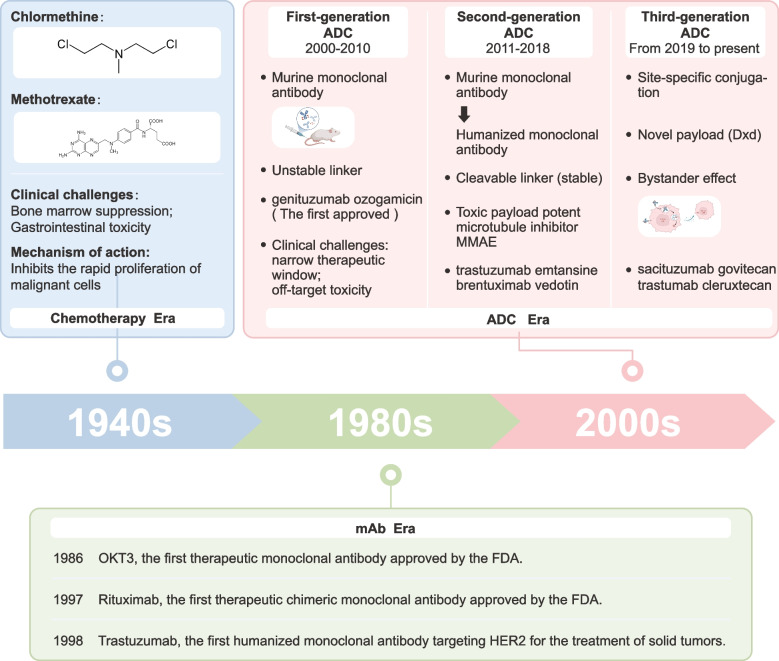
Timeline and evolution of anticancer therapies from chemotherapy to third-generation ADCs. A schematic overview depicting the historical evolution of anticancer treatment modalities. Early cytotoxic chemotherapy agents, such as chlormethine and methotrexate introduced in the 1940 s, marked the beginning of modern oncology. The 1980 s witnessed the advent of therapeutic mAbs, initiating the mAb era. Since 2000, the development of ADCs has progressed through three generations. First-generation ADCs, exemplified by gemtuzumab ozogamicin, were limited by unstable linkers and off-target toxicity. Second-generation ADCs incorporated cleavable linkers and potent cytotoxic payloads, such as MMAE. Third-generation ADCs, characterized by site-specific conjugation and bystander effects, have improved therapeutic indices through enhanced efficacy and reduced systemic toxicity. Representative agents from each generation are highlighted.

Despite notable advances in early diagnosis and conventional therapies, cancer remains a leading global health burden, accounting for over 10 million deaths annually [1–3]. Over time, cancer treatment has evolved from nonspecific cytotoxic chemotherapy to molecularly targeted strategies that aim to maximize therapeutic efficacy while minimizing systemic toxicity (Fig. 1). Traditional chemotherapeutic agents, such as alkylating agents and antimetabolites, introduced in the mid-twentieth century, became foundational to cancer therapy by exploiting the rapid proliferation of malignant cells [4–7]. However, their limited selectivity often results in substantial off-target toxicities, including myelosuppression, gastrointestinal damage, and organ injury, underscoring the urgent need for more precise treatment approaches [8–10]. The introduction of monoclonal antibodies (mAbs) in the 1980 s enabled the selective targeting of tumor-associated antigens, exemplified by trastuzumab (targeting HER2) and rituximab (targeting CD20). This innovation significantly improved clinical outcomes in subsets of hematologic malignancies and solid tumors, marking a paradigm shift in cancer therapy [11, 12]. Nevertheless, despite their clinical success, mAbs as monotherapies have shown limited efficacy in complex solid tumors, largely due to antigen heterogeneity, inadequate tumor penetration, and intrinsic resistance mechanisms [13, 14].

To address these limitations, antibody-drug conjugates (ADCs) have emerged as a promising class of therapeutics, combining the target specificity of mAbs with the high cytotoxic potency of chemotherapeutic agents. By selectively delivering cytotoxic payloads to tumor cells, ADCs aim to enhance antitumor activity while reducing systemic toxicity [15, 16]. A pivotal milestone in this field was achieved in 2000 with the approval of gemtuzumab ozogamicin (Mylotarg®), the first ADC approved for clinical use. This landmark event ushered in a new era of “biological missiles”, in which potent cytotoxic agents are directed specifically to cancer cells, minimizing damage to healthy tissues [17, 18]. Over the past two decades, ADC technology has progressed through three generations, evolving from early proof-of-concept to programmable precision therapeutics [19]. The first generation (2000-2010), represented by gemtuzumab ozogamicin, used humanized antibodies randomly conjugated to cytotoxins like calicheamicin, but suffered from unstable linkers, variable drug-to-antibody ratios (DARs), and poor payload control, leading to narrow therapeutic windows and off-target toxicity [20, 21]. The second generation (2011–2018) introduced humanized antibodies, cleavable linkers, and engineered conjugation sites, improving DAR consistency (typically ~3-4) and enabling the use of potent microtubule inhibitors like MMAE and DM1. This led to successful agents such as brentuximab vedotin (Adcetris®) and trastuzumab emtansine (Kadcyla®) [22–24]. The third generation (2019-present) has focused on site-specific conjugation, tumor-activated linkers, and next-generation payloads like topoisomerase I inhibitors (DXd, SN-38), which support both targeted cytotoxicity and a bystander effect [25, 26]. Notable examples include trastuzumab deruxtecan (Enhertu®) and sacituzumab govitecan (Trodelvy®) [27]. Across all generations, the overarching goal remains: to improve tumor selectivity, reduce toxicity, and overcome resistance.

Despite these advancements, ADCs continue to face substantial clinical hurdles. Resistance mechanisms include target antigen loss or mutation, impaired internalization, lysosomal dysfunction, and upregulation of drug efflux transporters, all of which reduce intracellular drug concentrations and diminish efficacy [28–30]. Toxicity remains a key challenge, particularly on-target effects in normal tissues (e.g., HER2-associated cardiotoxicity) and off-target adverse events stemming from payload diffusion, such as myelosuppression induced by topoisomerase inhibitors [31–33]. In solid tumors, high interstitial pressure, poor tissue penetration, antigen heterogeneity, and a lack of predictive biomarkers further hinder ADC delivery and patient stratification. Addressing these limitations will require continued innovation in molecular design, including bispecific ADCs, smart linker systems, and non-classical payloads, as well as integration into synergistic combination regimens, particularly with immunotherapies or microenvironment-modulating agents [34]. Together, these efforts represent the next frontier in unlocking the full therapeutic potential of ADCs across diverse and challenging cancer types.

To overcome current limitations and expand therapeutic potential, next-generation ADC platforms are reshaping drug design. Bispecific ADCs that co-target antigens such as HER2 and EGFR address tumor heterogeneity and improve targeting precision [35–37]. Immune-stimulatory ADCs, using payloads like TLR8 or STING agonists, combine cytotoxicity with immune modulation [38, 39]. Proteolysis-targeting chimeras (PROTAC)-based ADCs, which induce degradation of intracellular targets via the ubiquitin-proteasome system, offer strategies for traditionally “undruggable” proteins [40]. Meanwhile, novel payloads, including IDO inhibitors, PARP inhibitors, and epigenetic modulators, are expanding ADC functions beyond cytotoxicity toward immune activation and tumor reprogramming [41, 42]. These advances enable multi-mechanistic synergy, enhance selectivity, reverse resistance, and support rational combinations with checkpoint inhibitors [43]. Collectively, they represent a paradigm shift from conventional cytotoxic agents to a novel class of complex, precision-designed tumor-targeted platforms.

While numerous reviews have extensively detailed the structural features and clinical applications of ADCs [18, 43], there remains a significant gap in integrative analyses that comprehensively link their pharmacologic foundations with translational challenges, emerging design strategies, and future research trajectories. Despite recent advances, many patients continue to face limited therapeutic options, particularly in tumors with low antigen expression, acquired resistance, or immune-evasive microenvironments. Distinctively, this Review provides a cross-disciplinary perspective that integrates structural biology, pharmacology, and tumor immunology with insights from adjacent fields such as AI-guided design and nanotechnology-enabled delivery. By aligning molecular innovation with pressing clinical imperatives, we delineate a strategic roadmap to expedite translational progress and optimize therapeutic outcomes for patients receiving advanced ADC therapies.

## Basic structure and mechanism of ADCs

ADCs are a class of precisely engineered biopharmaceuticals that synergistically combine the target specificity of mAbs with the potent cytotoxicity of small-molecule chemotherapeutic agents [37, 43, 44]. Structurally, ADCs comprise three essential components: a monoclonal antibody, a chemical linker, and a highly potent cytotoxic payload. The coordinated interaction among these elements is critical to determining the overall therapeutic efficacy of the conjugate [45, 46]. This section provides a comprehensive overview of the structural and mechanistic foundations of ADCs, focusing on three key components: antibody design and antigen recognition, linker chemistry and stability, and the selection and evolution of cytotoxic payloads.

### Antibody component: antigen recognition and optimization

The antibody moiety serves as a tumor-targeting vector, enabling selective delivery of cytotoxic payloads to malignant cells while sparing normal tissues [20, 47, 48]. Most clinically approved ADCs utilize humanized or fully human IgG1 antibodies, which offer extended serum half-lives through neonatal Fc receptor (FcRn)-mediated recycling and preserve effector functions such as antibody-dependent cellular cytotoxicity (ADCC) and complement activation [20, 48]. An optimal tumor-associated antigen typically fulfills three criteria: (a) high and homogeneous expression on malignant cells with minimal presence in normal tissues, (b) efficient internalization upon antibody binding to facilitate intracellular drug delivery, and (c) biological relevance to tumorigenesis, which reduces immune escape and supports durable therapeutic responses [48–50]. Clinically validated targets satisfying these criteria include HER2 [51, 52], CD30 [53, 54], CD33 [55, 56], and TROP2 [57, 58], which have been successfully utilized in approved ADC therapies. While conventional monospecific antibodies currently dominate ADC design, structural optimization strategies are gaining traction. Biparatopic antibodies, which recognize two distinct epitopes on the same antigen, exhibit enhanced binding avidity and improved internalization kinetics of antibody-antigen complexes [59–61]. In parallel, advances in antibody engineering now allow site-specific conjugation and precise control over DAR, improving batch uniformity and maximizing the therapeutic index of ADCs [62, 63]. Although bispecific antibodies (BsAbs) have demonstrated clinical potential in T cell redirection strategies, such as CD3-EGFR constructs, their systematic integration into ADC platforms remains largely unexplored, with applications currently limited to early-stage investigations [64–69]. Accordingly, ADC development continues to favor monospecific antibodies due to their well-characterized pharmacokinetic behavior and predictable biodistribution [70, 71].

### Linker design: balancing stability and payload release

The linker component plays a dual role in ADC function: maintaining conjugate stability in systemic circulation and enabling efficient payload release within tumor cells. An ideal linker remains intact in plasma to prevent premature cleavage, yet is selectively cleaved in the tumor microenvironment (TME) to enable targeted drug release [21, 72]. Linker physicochemical properties, such as hydrophilicity and charge, substantially affect solubility, systemic stability, volume of distribution, and clearance kinetics, thereby influencing efficacy, safety, and the therapeutic index [73–76]. Hydrophilic linkers can improve aqueous solubility, reduce hydrophobic aggregation, and minimize nonspecific protein interactions, collectively extending plasma half-life and enhancing tissue distribution. Conversely, highly hydrophobic linkers may promote aggregation and rapid systemic clearance [20, 77]. Incorporating hydrophilic polyethylene glycol (PEG) chains into linker structures has been shown to improve blood stability and prolong circulation time [78]. Linker charge is likewise critical: negatively charged or neutral linkers generally confer greater plasma stability and reduced nonspecific tissue uptake, whereas positively charged linkers may increase hepatic and renal accumulation due to electrostatic interactions with cell membranes, potentially exacerbating off-target toxicity [18]. Moreover, residual chemical groups left on the payload after linker cleavage can alter membrane permeability, thereby influencing payload diffusion within the tumor and modulating the “bystander effect” [79].

Linkers are typically categorized as either cleavable or non-cleavable, depending on whether they are designed to respond to specific physiological conditions (Table 1). Cleavable linkers exploit tumor-specific biochemical cues, such as protease activity (e.g., cathepsin B-sensitive dipeptides) [20, 69], reductive intracellular environments (e.g., glutathione-sensitive disulfide bonds) [80–82], or acidic pH [83, 84], to achieve conditional drug release. These linkers generally exhibit efficient intracellular payload liberation, enabling “on-demand” release that enhances selectivity and therapeutic index [85]. However, some cleavable linkers lack sufficient stability in systemic circulation, leading to premature drug release and off-target toxicity, which was an issue that plagued early-generation ADCs [21, 86]. In contrast, non-cleavable linkers, such as thioether-based structures, rely on lysosomal degradation of the antibody component to release the payload, thereby enhancing in vivo stability and reducing systemic toxicity [87]. Nonetheless, this approach may limit the “bystander effect”, especially when the payload lacks the membrane permeability required for diffusion into neighboring antigen-negative tumor cells [78]. Recent advances in linker chemistry have driven the development of “smart” or stimuli-responsive linkers, which enable precise drug release in response to multiple tumor-specific triggers, including acidic pH, tumor-associated enzymes, and reactive oxygen species (ROS) [88]. These next-generation linkers are tailored to the distinct features of the TME, expanding the therapeutic window while reducing systemic exposure and off-target effects [89].

**Table 1:** Comparison of different linker types used in ADCs

Linker Type		Cleavage mechanism	Examples	Advantages	Limitations
Cleavable	Acid-labile	Low pH	Hydrazone	Simple design; effective in acidic microenvironment	Poor plasma stability; off-target release
	Enzyme-cleavable	Protease-sensitive	Val-Cit, Phe-Lys, Ala-Ala	Tumor-selective; widely validated clinically	Potential for premature release in inflamed tissues
	Disulfide	Intracellular glutathione-mediated reduction	Disulfide bonds	Responsive to tumor cytosol; rapid release	Variable stability; sensitive to systemic redox changes
	β-glucuronide	β-glucuronidase	β-glucuronide linker	Tumor-specific release; low background activity; promising in preclinical studies	Limited clinical validation; synthetic complexity; early-stage development
	Photo-cleavable	Light activation	o-Nitrobenzyl derivatives	Precise spatiotemporal control of drug release; useful for mechanistic studies; promising in controlled environments	Limited light penetration in tissues; requires external activation; currently limited to preclinical research
Non-cleavable		Lysosomal degradation of antibody	Thioether (SMCC), PEG-based	Excellent plasma stability; reduced systemic toxicity	Requires full antibody degradation for payload release

### Cytotoxic payloads: from classical toxins to targeted agents

Cytotoxic payloads are the core components of ADCs responsible for direct tumor cell killing [90]. Their design must strike a balance between ultra-high potency, sufficient chemical stability, and favorable physicochemical properties [91, 92]. Given the limited number of ADC molecules that successfully reach the intracellular compartment of tumor cells, the payload must exhibit cytotoxic activity at sub-nanomolar or even picomolar concentrations to ensure therapeutic efficacy with minimal exposure [47, 93, 94]. Currently, the most widely used and studied payload classes fall into two main categories: microtubule inhibitors and DNA-damaging agents [95]. Microtubule-disrupting agents, such as monomethyl auristatin E (MMAE) [95, 96], monomethyl auristatin F (MMAF) [97, 98], and maytansinoids (DM1, DM4) [99], induce mitotic arrest and cell death [45, 100]. DNA-damaging agents, including calicheamicin [101], duocarmycins [102], and pyrrolobenzodiazepine (PBD) dimers [103, 104], exert their effects by inducing DNA strand breaks or alkylation, thereby triggering apoptosis [105, 106]. These highly potent agents were extensively employed in first-generation ADCs due to their rapid and potent cytotoxic activity [107]. However, their clinical application has been limited by a narrow therapeutic window and susceptibility to drug resistance [93, 108].

To overcome the limitations of traditional mechanisms of action, small-molecule inhibitors of topoisomerase I have been incorporated into next-generation ADCs [109]. Notably, deruxtecan (used in trastuzumab deruxtecan, T-DXd) and SN-38 (used in sacituzumab govitecan, Trodelvy) are representative examples of enzyme inhibitors with both potent cytotoxicity and membrane permeability [110]. These features enable a “bystander effect”, allowing the elimination of neighboring tumor cells with low or heterogeneous antigen expression and broadening the clinical utility of ADCs [57]. Although traditional cytotoxic agents remain the primary focus, recent research has started to explore the potential of incorporating small-molecule inhibitors as functional payloads in ADC therapies, such as PARP inhibitors [111, 112], CDK inhibitors [113, 114], and immune-modulating agents [115–117]. These novel payloads have the potential to offer targeted therapies with reduced systemic toxicity and enhanced efficacy, especially against drug-resistant tumors. However, challenges related to stability, intracellular release, and clinical validation remain [118]. Despite these obstacles, the potential use of functional small molecules as ADC payloads is an emerging area of interest for future research and development [119] (Table 2).

**Table 2:** Comparison of cytotoxic payloads used in ADCs

Payload class	Mechanism of action	Representative payloads	Advantages	Limitations
Microtubule inhibitors	Inhibit tubulin polymerization, mitotic arrest → apoptosis	MMAE, MMAF, DM1, DM4	Well-characterized; fast-acting; effective in solid and hematologic tumors	Peripheral neuropathy; multidrug resistance risk
DNA-damaging agents	DNA alkylation or cleavage → apoptosis	Calicheamicin, Duocarmycin, PBD dimers	Extremely potent; irreversible DNA damage	Narrow therapeutic window; off-target genotoxicity
Topoisomerase I inhibitors	Inhibit DNA replication by blocking topoisomerase I	SN-38, Deruxtecan	Bystander effect; effective in heterogenous tumors	Moderate toxicity; interstitial lung disease (rare)

### Mechanisms of action: from target recognition to tumor eradication

**Fig. 2:**
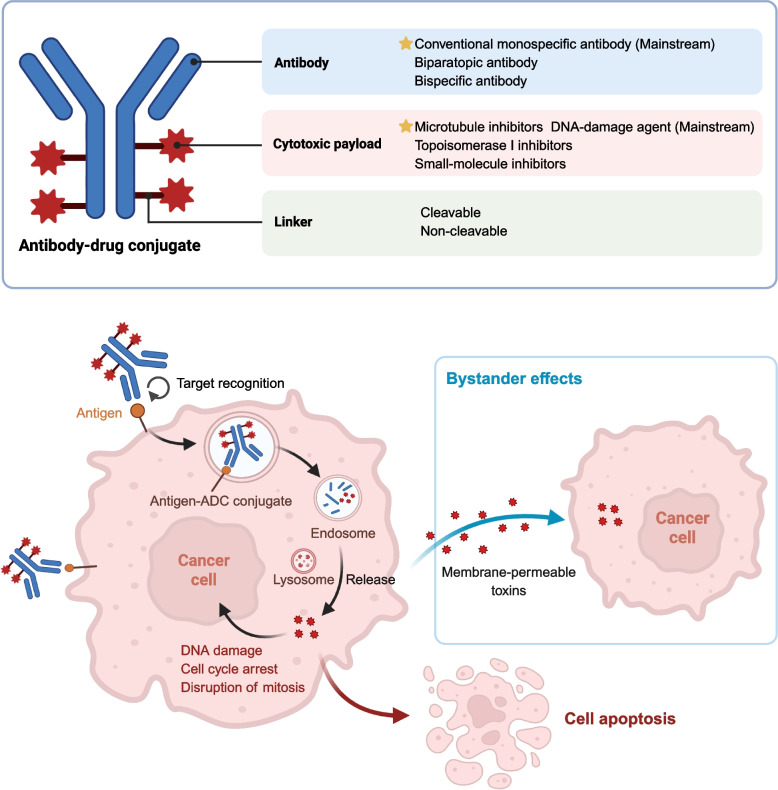
Structural components and mechanisms of action of ADCs. ADCs comprise three essential components: a mAb for tumor-specific targeting, a cytotoxic payload for tumor cell killing, and a chemical linker that governs drug release. Upon binding to tumor-associated surface antigens, the ADC-antigen complex is internalized via endocytosis, trafficked to endosomes and lysosomes, and undergoes intracellular release of the cytotoxic agent. This release induces cell death through mechanisms such as DNA damage, mitotic disruption, and cell cycle arrest. Importantly, membrane-permeable payloads may diffuse into adjacent antigen-negative tumor cells, producing a bystander effect that enhances efficacy in heterogeneous tumors.

The therapeutic efficacy of ADCs is orchestrated through a cascade of interdependent molecular events that span from extracellular antigen engagement to intracellular execution of cytotoxic activity (Fig. 2). These mechanisms collectively define not only the antitumor potential of ADCs but also shape their pharmacokinetic properties, safety margins, and vulnerability to resistance. The principal modes of action include antigen-specific binding, internalization, intracellular drug release, disruption of essential cellular pathways, and in some cases, immunomodulatory effects [47, 120]. Therapeutic action is initiated by the selective binding of ADCs to tumor-associated surface antigens, which triggers clathrin-mediated endocytosis and intracellular trafficking through the endolysosomal pathway [120, 121]. Within lysosomes, the intracellular milieu characterized by proteolytic enzymes, acidic pH, or reductive redox conditions facilitates payload release, contingent on the design of the chemical linker. Cleavable linkers enable stimulus-responsive drug liberation, whereas non-cleavable linkers necessitate complete proteolytic degradation of the antibody backbone for payload activation [120, 122]. Following release, the cytotoxic payload translocates to its intracellular site of action, eliciting cellular demise through various mechanisms. DNA-damaging agents, such as topoisomerase I inhibitors and DNA alkylators, induce replication stress, chromatin destabilization, and double-strand breaks, culminating in the activation of DNA damage checkpoints, G2/M arrest, and apoptotic cell death [24, 123]. Microtubule-disrupting agents, on the other hand, perturb mitotic spindle dynamics, leading to mitotic catastrophe. In tumors with heterogeneous or low antigen expression, membrane-permeable payloads, including SN-38 and deruxtecan, are capable of diffusing into adjacent antigen-negative cells, thereby mediating a “bystander effect” that amplifies therapeutic coverage across spatially diverse tumor cell populations [47, 122, 124].

In addition to direct cytotoxicity, several ADCs have demonstrated the capacity to induce immunogenic cell death (ICD), a regulated form of cell demise characterized by the emission of damage-associated molecular patterns (DAMPs) such as ATP, HMGB1, and surface-exposed calreticulin [125]. These immunologic signals promote dendritic cell recruitment and maturation, enhance tumor antigen cross-presentation, and prime adaptive immune responses. The immunostimulatory potential of ADCs has emerged as a mechanistic rationale for their combinatorial use with immune checkpoint inhibitors and other immunotherapeutic modalities [126, 127]. Collectively, these mechanistic dimensions underscore the unique therapeutic architecture of ADCs as rationally engineered agents that integrate selective tumor targeting, intracellular payload activation, and, when applicable, immune modulation. A refined understanding of these interconnected processes remains essential for overcoming resistance, optimizing therapeutic indices, and expanding the clinical applicability of ADCs across oncologic indications. 

### Clinical applications of ADCs

As a breakthrough in precision oncology, ADCs have demonstrated substantial clinical translational value in both solid tumors and hematologic malignancies in recent years [15]. By combining targeted delivery with potent cytotoxicity, several ADCs have successfully transitioned from salvage settings to first-line and even neoadjuvant indications [24, 43]. More importantly, they have achieved historic improvements in survival outcomes for patients with tumors refractory to conventional therapies [85]. As of 2025, a total of 15 ADCs have received FDA approval, collectively covering more than 15 distinct tumor subtypes, according to the Drugs@FDA database, although one of them has been withdrawn from the market (https://www.accessdata.fda.gov/scripts/cder/daf). Meanwhile, over 1300 clinical trials are actively ongoing worldwide according to an industry report, reflecting the rapid expansion and growing interest in this therapeutic modality (https://www.precisionformedicine.com/blog/clinical-trial-trends-antibody-drug-conjugates). This section provides a comprehensive overview of the clinical development landscape of ADCs, focusing on four key aspects: approved agents, therapeutic efficacy and safety profiles, cutting-edge investigational pipelines, and combination treatment strategies.

### Approved ADCs: target diversity and indication expansion

**Fig. 3:**
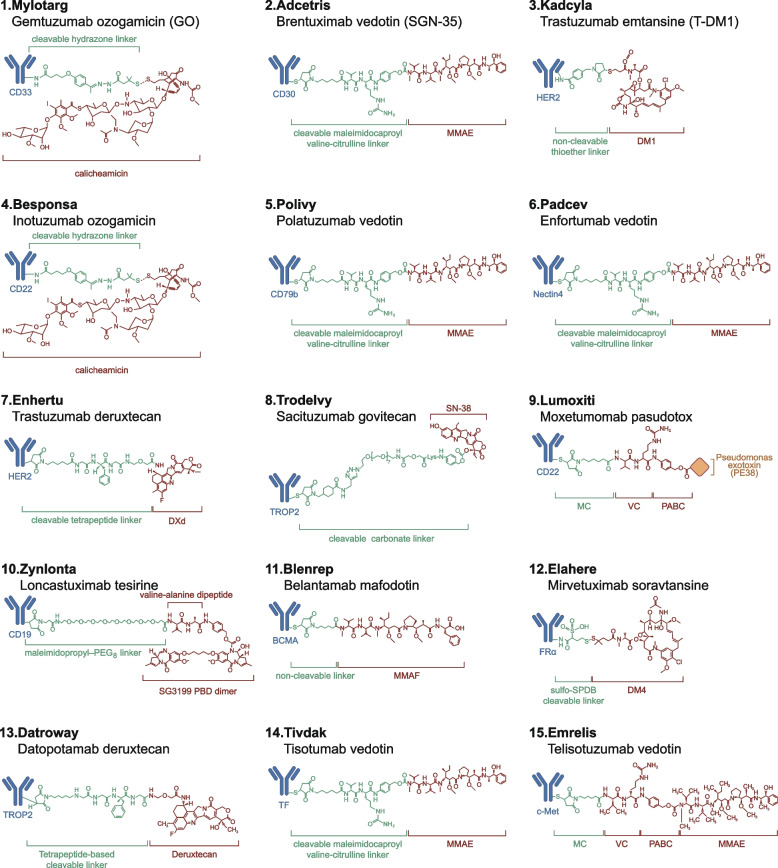
Structural overview of FDA-approved ADCs. Structural features of 15 FDA-approved ADCs are presented, encompassing their antigen targets, antibody backbones, cytotoxic payloads, and linker chemistries. These ADCs employ a range of payload classes, including calicheamicin, MMAE, DM1, DXd, SN-38, and PBD dimers, conjugated via cleavable or non-cleavable linkers. Target antigens include CD33, HER2, TROP2, and TF, among others. Variations in these structural components influence internalization efficiency, drug release mechanisms, pharmacokinetics, and antitumor efficacy, reflecting the molecular complexity and functional diversity of approved ADCs.

Since the approval of the first ADC, gemtuzumab ozogamicin (Mylotarg®), targeting CD33 for acute myeloid leukemia (AML) in 2000, the global ADC landscape has expanded considerably [128, 129]. To date, a total of 15 ADCs have received regulatory approval, covering a broad spectrum of malignancies, including breast cancer, gastric cancer, non-small cell lung cancer (NSCLC), ovarian cancer, cervical cancer, urothelial carcinoma, Hodgkin lymphoma, diffuse large B-cell lymphoma (DLBCL), and acute leukemia [56, 130, 131] (Fig. 3, Table 3). The evolution of ADC target selection has progressed from hematologic-specific CD antigens (e.g., CD30, CD22, CD33, CD79b) to tumor-associated antigens more commonly expressed in solid tumors, such as HER2, TROP2, Nectin-4, and folate receptor α [20, 47]. This shift has enabled a “pan-cancer, broad-coverage” therapeutic strategy. In terms of payload design, there has been a transition from classical microtubule inhibitors (e.g., MMAE, DM1) to DNA-damaging agents (e.g., calicheamicin, PBD) and topoisomerase I inhibitors (e.g., SN-38, deruxtecan) [99, 104, 110, 132]. This reflects a broader paradigm shift in ADC development, from simple cytotoxic delivery vehicles to mechanistically diverse tumor-targeted therapies. For example, T-DXd was the first ADC approved for HER2-low breast cancer based on immunohistochemical weak positivity [110, 133], while sacituzumab govitecan (Trodelvy®) showed remarkable activity in heavily pretreated triple-negative breast cancer (TNBC), supporting the value of ADCs in patient populations with limited therapeutic options [134, 135].

**Table 3:** FDA-approved ADCs with indications, approval dates, clinical efficacy, and toxicities

Drug (Brand name)	Target	Indications	Approval date	Clinical efficacy	Major toxicities
Gemtuzumab ozogamicin (Mylotarg)	CD33	Acute myeloid leukemia	2000, 2017 (Re-approval)	Increased remission rates when combined with chemotherapy; improves ORR	Hepatic veno-occlusive disease, bone marrow suppression, infections, infusion-related reactions
Brentuximab vedotin (Adcetris)	CD30	Hodgkin lymphoma, Anaplastic large cell lymphoma, Peripheral T-cell lymphoma, Diffuse large B-cell lymphoma	2011	Significant improvement in ORR and PFS in relapsed or refractory patients	Peripheral neuropathy, hematologic toxicities, infections, gastrointestinal toxicity, infusion-related reactions
Trastuzumab emtansine (Kadcyla)	HER2	HER2-positive metastatic breast cancer	2013	Approximately 5.8 months improvement in OS; significant DFS improvement	Hepatotoxicity, cardiotoxicity, fatigue, peripheral neuropathy, infusion-related reactions
Inotuzumab ozogamicin (Besponsa)	CD22	Acute lymphoblastic leukemia	2017	Improved ORR compared to standard chemotherapy, particularly in relapse or refractory patients	Hematologic toxicity, hepatic toxicity, infections, gastrointestinal toxicity, infusion-related reactions
Polatuzumab vedotin (Polivy)	CD79b	Diffuse large B-cell lymphoma	2019	Significant improvement in ORR with higher PFS	Hematologic toxicity, peripheral neuropathy, fatigue, infections, gastrointestinal toxicity, infusion-related reactions
Enfortumab vedotin (Padcev)	NECTIN-4	Urothelial carcinoma	2019	Significant improvement in ORR, especially in resistant disease	Skin reactions, gastrointestinal toxicity, hematologic toxicity, infusion-related reactions
Trastuzumab deruxtecan (Enhertu)	HER2	HER2-positive breast cancer, gastric cancer, non-small cell lung cancer	2019	Significant improvement in PFS and OS, especially in HER2- positive breast cancer	Interstitial lung disease, hematologic toxicity, gastrointestinal toxicity, skin reactions, fatigue, infusion-related reactions
Sacituzumab govitecan (Trodelvy)	TROP2	Triple-negative breast cancer	2020	Significant improvement in PFS and OS for triple-negative breast cancer patients	Hematologic toxicity, diarrhea, fatigue, nausea and vomiting, anemia and thrombocytopenia, infusion-related reactions
Belantamab mafodotin (Blenrep)	BCMA	Multiple myeloma	2020, withdrawn in 2022	Significant improvement in ORR, especially in relapsed or refractory patients	Ocular toxicity, hematologic toxicity, infections, fatigue, gastrointestinal toxicity, infusion-related reactions
Moxetumomab pasudotox (Lumoxiti)	CD22	Hairy cell leukemia	2018	Significant remission rate with remarkable efficacy	Hematologic toxicity, renal toxicity, hemorrhagic events, fatigue
Loncastuximab tesirine (Zynlonta)	CD19	Diffuse large B-cell lymphoma	2021	Significant improvement in ORR and PFS	Hematologic toxicity, infections, fatigue, elevated liver enzymes, peripheral edema
Mirvetuximab soravtansine (Elahere)	FRα	Ovarian cancer	2022	Significant improvement in PFS and ORR, particularly in platinum-resistant patients	Ocular toxicity, gastrointestinal issues, hepatotoxicity, fatigue, skin reactions, infusion-related reactions
Datopotamab deruxtecan (Datroway)	TROP2	HR-positive, HER2-negative breast cancer; EGFR-mutated non-small cell lung cancer	2025	Significant improvement in PFS and ORR in several TROP2-positive solid tumors	Interstitial lung disease, hematologic toxicity, gastrointestinal toxicity, ocular toxicity, fatigue
Tisotumab vedotin (Tivdak)	TF	Cervical cancer	2021	Significant improvement in ORR and PFS, particularly in recurrent or metastatic cervical cancer	Ocular toxicity, gastrointestinal issues, hepatotoxicity, fatigue, hematologic toxicity, peripheral neuropathy, skin reactions, infusion-related reactions
Telisotuzumab vedotin (Emrelis)	c-MET	Non-small cell lung cancer	2025	Significant improvement in PFS, particularly in EGFR-mutant NSCLC patients	Ocular toxicity, gastrointestinal issues, hepatotoxicity, fatigue, hematologic toxicity, peripheral neuropathy

### Efficacy and safety: clinical gains and toxicity risks

Clinical data from pivotal trials demonstrate that ADCs have achieved superior efficacy compared to standard chemotherapy or targeted therapies across multiple tumor types [56, 136]. For instance, T-DXd significantly improved progression-free survival (PFS) and overall survival (OS) in patients with HER2-low breast cancer in the DESTINY-Breast04 trial, extending median PFS from 5.1 to 9.9 months and OS from 16.8 to 23.4 months, with an objective response rate (ORR) of 52.6% [110]. Similarly, sacituzumab govitecan (Trodelvy®) achieved a median PFS of 5.6 months and an ORR of 35% in the ASCENT study for refractory TNBC, outperforming conventional chemotherapy [135]. Encouraging results have also been reported for ADCs in other tumor types such as NSCLC, gastric cancer, and bladder cancer, where several agents have matched or exceeded the efficacy of existing therapies [137–139]. However, the clinical success of ADCs is often accompanied by a distinctive spectrum of adverse events associated with their highly potent cytotoxic payloads. Common toxicities include myelosuppression, hepatotoxicity, interstitial lung disease (ILD), and ocular complications, which are influenced by factors such as payload mechanism of action, linker stability, and antigen distribution in normal tissues [140–144]. For example, T-DXd, which utilizes a topoisomerase I inhibitor, has been associated with ILD in several trials, including cases with fatal outcomes [145–147]. Trodelvy frequently causes neutropenia and diarrhea due to its SN-38 payload [148], while MMAE-containing ADCs have been linked to peripheral neuropathy [149]. Additionally, on-target off-tumor toxicity may occur when the target antigen is expressed at low levels in healthy tissues, as observed with Nectin-4 in the urothelium, leading to cutaneous and ocular side effects [150, 151]. To enhance the therapeutic index of ADCs, ongoing research efforts focus on improving linker stability, reducing premature systemic payload release, optimizing the DAR, and selecting targets with high tumor specificity and efficient internalization [73, 152] Moreover, the integration of companion diagnostics (CDx) is facilitating the shift from simple “target-positive” patient selection toward quantitative, expression-driven stratification, thereby improving clinical precision and benefit-to-risk balance [153, 154].

### Clinical development: structural innovation and indication expansion

**Fig. 4:**
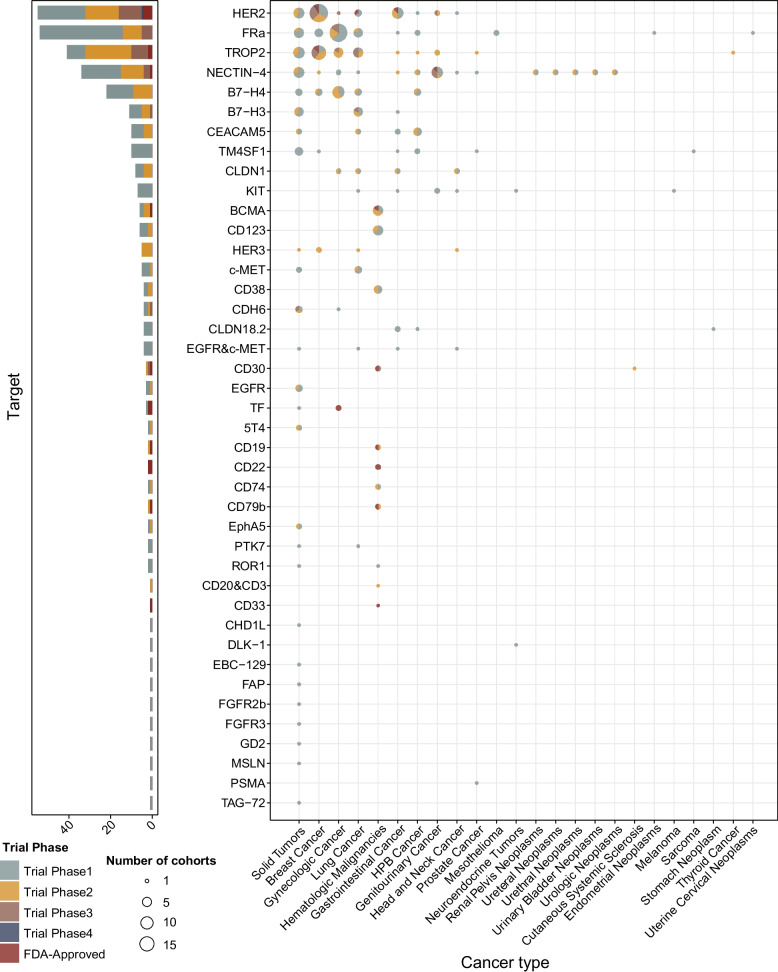
Target-indication landscape of ADCs in clinical trials and approved therapies. The target-indication landscape of ADCs spans a broad range of tumor-associated antigens across both approved and investigational agents. Established targets such as HER2, TROP2, FRα, and Nectin-4 are broadly expressed in solid tumors, including breast, lung, gynecologic, and genitourinary malignancies. In parallel, emerging antigens, including HER3, B7-H3, CEACAM5, TM4SF1, KIT, and B7-H4, are under active investigation for their therapeutic relevance, particularly in genitourinary, HPB, and breast cancers. Dual-targeting ADCs, such as those co-engaging EGFR and c-MET or CD20 and CD3, are being developed to overcome tumor heterogeneity and improve targeting specificity.

**Fig. 5:**
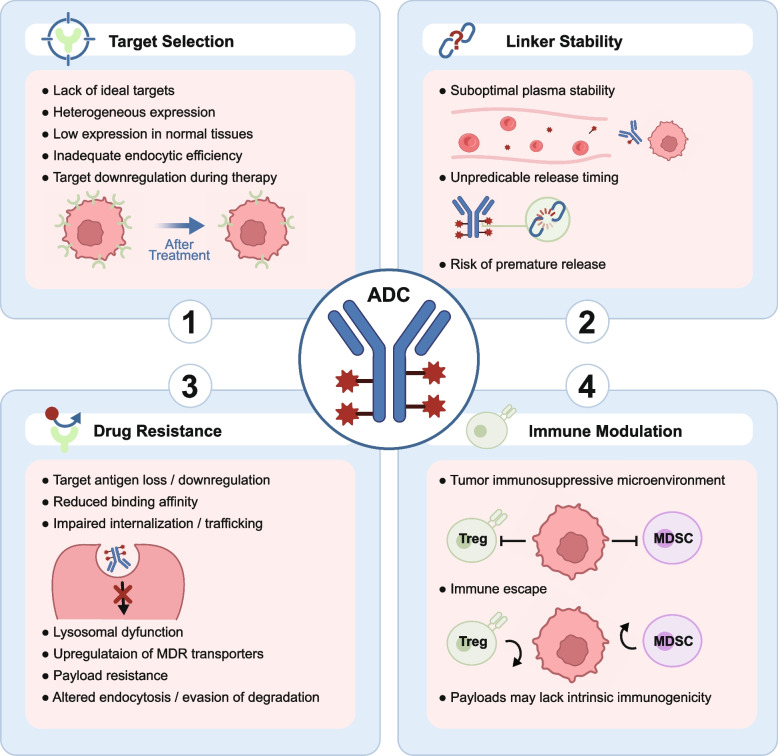
Key challenges limiting the efficacy of ADCs. Multiple challenges constrain the clinical performance of ADCs. (1) Target selection is hindered by antigen heterogeneity, low tumor-specific expression, inefficient endocytosis, and dynamic downregulation during therapy. (2) Linker stability issues, including suboptimal plasma half-life and premature payload release, compromise therapeutic index. (3) Drug resistance mechanisms include antigen loss, impaired internalization, lysosomal dysfunction, efflux transporter upregulation, and payload-specific resistance. (4) Immune modulation within the TME-mediated by Tregs, MDSCs, and immune escape mechanisms, can dampen ADC-induced antitumor responses, particularly when the payload lacks intrinsic immunogenicity.

The global pipeline of ADCs is expanding rapidly. According to a report by Precision for Medicine, over 1300 active clinical trials are currently underway, and more than 100 unique antigen targets are under investigation. These trials span a wide range of development stages, from early-phase explorations to pivotal phase III studies, and aim to validate novel targets, enhance payload diversity, and optimize delivery strategies [155]. Notably, this figure may differ from counts in public databases such as ClinicalTrials.gov, which lists fewer trials due to variations in registration scope and classification criteria. Among the most intensively studied targets are HER2, TROP2, FRα, and Nectin-4, which have yielded multiple approved agents and late-stage clinical trials across breast, lung, urothelial, and ovarian cancers. In parallel, emerging antigens such as HER3 [156], B7-H3 [157, 158], CEACAM5 [159, 160], TM4SF1 [161, 162], KIT [163, 164], and B7-H4[165, 166] have become focal points of current research, particularly in tumor types with limited treatment options, including genitourinary cancer, breast cancer, hepatopancreatobiliary (HPB) cancer, lung cancer, and neuroendocrine neoplasms (Fig. 4, Table 4). For example, patritumab deruxtecan, a HER3-directed ADC, has shown promising efficacy in EGFR-mutated NSCLC [167], while tusamitamab ravtansine, targeting CEACAM5, has demonstrated anti-tumor activity in preclinical models and early-phase studies [168, 169]. Several investigational ADCs have entered late-stage clinical development and are demonstrating potential breakthrough activity. Datopotamab deruxtecan (Dato-DXd), a TROP2-targeted ADC with a topoisomerase I payload, is undergoing head-to-head phase III trials versus chemotherapy in both triple-negative and hormone receptor-positive/HER2-negative breast cancer [170, 171]. MGC018, directed against B7-H3, has shown preliminary antitumor activity in metastatic castration-resistant prostate cancer [158, 172, 173], while other candidates such as trastuzumab duocarmazine (targeting HER2) [174] and ABBV-400 (targeting CEACAM5) [175] are progressing through phase II/III studies. Although the systematic integration of BsAbs into ADC platforms remains largely unexplored, several innovative constructs, such as bispecific ADCs, new payload ADCs, and activatable linkers, have now entered early clinical evaluation of several prototype designs, indicating a shift toward broader structural innovation in the field [176–178]. A representative example is camidanlumab tesirine, a CD25-targeted ADC using a PBD dimer payload, which has demonstrated durable responses in relapsed/refractory Hodgkin lymphoma [179–181]. This new generation of ADCs exemplifies a shift from traditional cytotoxic payload delivery to mechanistically tailored designs that integrate advanced antibody engineering, smarter payloads, and disease-specific biomarkers. These innovations are expected to further expand the clinical impact of ADCs into resistant disease settings and previously untreatable malignancies (Fig. 5).

**Table 4:** Ongoing clinical trials of targeted therapies categorized by cancer type, molecular target, and trial phase

Number	Cancer type	Target
**Trial Phase1**		
NCT05824325	Breast Cancer	HER2
NCT06041516	Neuroendocrine Neoplasm	DLK-1
NCT06328387	Breast Cancer	HER2; TROP-2
NCT05308225	Multiple Myeloma	CD38
NCT05323045	Solid Tumor	MET
NCT05652868	Non-Small Cell Lung Cancer	MET
NCT06597721	Solid Tumor	TF
NCT05565807	Multiple Myeloma	CD38
NCT04982224	Non-Small Cell Lung Cancer	MET
NCT06625593	Solid Tumor	FGFR2b
NCT06238479	Solid Tumor; Bladder Cancer; Cervical Cancer; Head and Neck Squamous Cell Carcinoma; Solid Tumor; Non-Small Cell Lung Cancer; Ovarian Cancer; Pancreatic Cancer; Prostate Cancer; Recurrent Solid Tumor; Renal Pelvis Cancer	NECTIN-4
NCT05200364	Fallopian Tube Cancer; Ovarian Cancer; Primary Peritoneal Carcinoma	FRα
NCT05498597	Cancer; Carcinoma; Solid Tumor; Endometrial Adenocarcinoma; Endometrial Cancer; Endometrial Clear Cell Adenocarcinoma; Endometrial Endometrioid Adenocarcinoma; Endometrial Serous Adenocarcinoma; Lung Adenocarcinoma; Malignant Pleural Mesothelioma; Ovarian Cancer; Pancreatic Ductal Adenocarcinoma; Triple Negative Breast Cancer	FRα
NCT02099058	Solid Tumor	MET
NCT06805825	Adenoid Cystic Carcinoma; Chromophobe Renal Cell Carcinoma; Clear Cell Renal Cell Carcinoma; Gastrointestinal Stromal Tumors; Neuroendocrine Tumors; Small-Cell Lung Cancer; Uveal Melanoma	KIT
NCT06710132	Gastric Cancer; Non-Small Cell Lung Cancer; Pancreatic Cancer; Pancreatic Ductal Adenocarcinoma; Solid Tumor	CEACAM5
NCT04039230	Breast Cancer	TROP-2
NCT06233942	Solid Tumor	B7-H4
NCT05527184	Cervical Cancer; Endometrial Cancer; Fallopian Tube Cancer; Ovarian Cancer; Primary Peritoneal Carcinoma	FRα
NCT05941507	Solid Tumors	TROP2
NCT05464030	Colorectal Cancer	CEACAM5
NCT06641908	Solid Tumor	GD2
NCT04822337	Multiple Myeloma	BCMA
NCT04189614	Cancer; Non-Small Cell Lung Cancer	PTK7
NCT06440005	Cancer; Angiosarcoma; Breast Cancer; Colorectal Cancer; Liver Cancer; Locally Carcinoma; Solid Tumor; Pancreatic Cancer; Prostate Cancer; Solid Tumor	TM4SF1
NCT06422520	Solid Tumor	B7H3
NCT06265727	Solid Tumor	NECTIN-4
NCT05980416	Digestive System Neoplasm; Gastrointestinal Neoplasms; Pancreas Neoplasm; Stomach Neoplasm	CLDN18.2
NCT06563804	Acute Myeloid Leukemi	CD74
NCT06525298	Solid Tumor	CHD1L
NCT06667960	Solid Tumor	5T4
NCT06747585	Cervical Squamous Cell Carcinoma; Esophageal Squamous Cell Carcinoma; Head and Neck Squamous Cell Carcinoma; Non-Small Cell Lung Cancer	CLDN1
NCT06774963	Solid Tumors; Biliary Tract Cancer; Breast Cancer; Endometrial Cancer; Non-Small Cell Lung Cancer; Ovarian Cancer	B7-H4
NCT05547321	Solid Tumor	FAP
NCT06234423	Ovarian Cancer; Solid Tumor	CDH6
NCT04300556	Solid Tumor	FRα
NCT06014658	Cancer	EphA5
NCT04450732	Solid Tumor; Salivary Gland Carcinomas; Biliary Tract Cancer; Breast Cancer	HER2
NCT06359002	Myelodysplastic Syndrome; Acute Myeloid Leukemi	CD123
NCT05948865	Cancer	EGFR
NCT06590857	Breast Cancer	HER2
NCT05872295	Breast Cancer; Gastric Cancer; Gastroesophageal Junction Cancer	HER2
NCT06400472	Non-Small Cell Lung Cancer; Colorectal Cancer; Endometrial Neoplasms; Ovarian Cancer; Pancreatic Neoplasm; Breast Cancer; Uterine Cervical Neoplasms	FRα
NCT05279300	Lymphoma; Solid Tumor	ROR1
NCT04042701	Breast Cancer; Non-Small Cell Lung Cancer	HER2
NCT06752681	Solid Tumor	PTK7
NCT06555744	Solid Tumor	FRα
NCT06057922	Solid Tumor	B7-H3
NCT05434234	Solid Tumor	B7-H3
NCT06362252	Small Cell Lung Cancer	B7-H3
NCT06244485	Solid Tumor	HER2; TROP-2
NCT04309981	Multiple Myeloma	BCMA
NCT05701527	Solid Tumor	EBC-129
NCT06959706	Solid Tumor	TAG-72
NCT05123482	Biliary Tract Carcinoma; Breast Cancer; Endometrial Cancer; Ovarian Cancer; Non-Small Cell Lung Cancer	B7-H4
NCT06336707	Solid Tumor	B7-H4
NCT06523803	Cancer	MSLN
NCT06384807	Solid Tumor	TROP-2
NCT05174637	Solid Tumor	TROP-2
NCT05688605	Solid Tumor	EGFR
NCT06825624	Colorectal Cancer	B7-H3
NCT06874335	Solid Tumor	FGFR3
NCT05647122	Solid Tumor; Non-Small Cell Lung Cancer; Colorectal Cancer; Head and Neck Neoplasms	EGFR × MET
NCT05564858	Solid Tumor	HER2
NCT04492488	Solid Tumor; Gastric Cancer; Gastroesophageal Junction Cancer	HER2
NCT06781983	Solid Tumor	NECTIN-4
NCT05511844	Breast Cancer	HER2
NCT04826341	Solid Tumor; Small Cell Lung Cancer	TROP-2
NCT05142189	Non-Small Cell Lung Cancer	B7-H3
NCT05143229	Breast Cancer	TROP-2
NCT04538742	Breast Cancer	HER2
NCT06121557	Breast Cancer	HER2
NCT04662580	Prostate Cancer	PSMA
NCT04556773	Breast Cancer	HER2
NCT03386513	Blastic Plasmacytoid Dendritic Cell Neoplasm; Myeloproliferative Neoplasm	CD123
NCT04686305	Non-Small Cell Lung Cancer	HER2
NCT03288545	Renal Pelvis Neoplasms; Ureteral Neoplasms; Urethral Neoplasms; Urinary Bladder Neoplasms; Urologic Neoplasms; Urothelial Cancer	NECTIN-4
NCT06827236	Breast Cancer	HER2
NCT05579366	Endometrial Cancer; Non-Small Cell Lung Cancer; Fallopian Tube Cancer; Ovarian Cancer; Breast Cancer; Mesothelioma; Primary Peritoneal Carcinoma; Breast Cancer; Uterine Cancer	FRα
**Trial Phase 2**		
NCT05979740	Bladder Cancer	HER2
NCT06263543	Breast Cancer	TROP2
NCT05824325	Breast Cancer	HER2; TROP2
NCT06328387	Breast Cancer	HER2; TROP2
NCT05308225	Multiple Myeloma	CD38
NCT06311214	Malignant Solid Neoplasm	NECTIN-4; HER2; TROP2
NCT05565807	Multiple Myeloma	CD38
NCT04982224	Non-Small Cell Lung Cance	c-MET
NCT05838521	Cervical Cancer	TROP2
NCT04251416	Endometrial Carcinoma	TROP2
NCT06710132	Non-Small Cell Lung Cancer; Pancreatic Cancer; Pancreatic Ductal Adenocarcinoma; Solid Tumor	CEACAM5
NCT04039230	Breast Cancer	TROP2
NCT05941507	Solid Tumor	TROP2
NCT04822337	Multiple Myeloma	BCMA
NCT06265727	Solid Tumor	NECTIN-4
NCT06563804	Acute Myeloid Leukemia	CD74
NCT06525298	Solid Tumor	HER2
NCT06667960	Solid Tumor	5T4
NCT06747585	Non-Small Cell Lung Cance; Cervical Squamous Cell Carcinoma; Esophageal Squamous Cell Carcinoma; Head and Neck Squamous Cell Carcinoma	CLDN1
NCT05280470	Small Cell Lung Cance	B7-H3
NCT06028932	Ovarian Cancer	TROP2
NCT04965766	Breast Cancer	HER2
NCT06918912	Lymphoma	CD19
NCT06555263	Non-Small Cell Lung Cance	FRα
NCT04300556	Solid Tumor	FRα
NCT06161025	Solid Tumor	CDH6
NCT06014658	Cancer	EphA5
NCT06590857	Breast Cancer	HER2
NCT06533826	Breast Cancer	HER2; TROP2
NCT06235216	Thyroid Cancer	TROP2
NCT04940325	Non-Small Cell Lung Cance	TROP2
NCT05798156	Lymphoma	CD79b
NCT06107686	Solid Tumor; Breast Cancer; Non-Small Cell Lung Cance; Head and Neck Squamous Cell Carcinoma	HER3
NCT06057922	Solid Tumor	B7-H3
NCT05613088	Ovarian Cancer	FRα
NCT06362252	Small Cell Lung Cance	B7-H3
NCT04309981	Multiple Myeloma	BCMA
NCT05123482	Biliary Tract Carcinoma; Breast Cancer; Endometrial Cancer; Ovarian Cancer; Non-Small Cell Lung Cancer	B7-H4
NCT06188559	Breast Cancer	HER2
NCT06649331	Breast Cancer	HER2; TROP2; HER3; NECTIN-4
NCT06384807	Solid Tumor	TROP2
NCT05064358	Multiple Myeloma	BCMA
NCT05493683	Colorectal Cancer	HER2
NCT06553885	Hepatocellular Carcinoma; Colorectal Cancer	NECTIN-4
NCT05688605	Solid Tumor	EGFR
NCT06014190	Endometrial Cancer; Fallopian Tube Cancer; Ovarian Cancer; Primary Peritoneal Cancer	B7-H4
NCT06112704	Solid Tumor	B7-H3
NCT05870748	Fallopian Tube Cancer; Ovarian Cancer; Primary Peritoneal Cancer	FRα
NCT04492488	Solid Tumors; Gastric Cancer; Gastroesophageal Junction Cancer;	HER2
NCT05410418	Lymphoma	CD20 × CD3
NCT04826341	Solid Tumors; Small Cell Lung Cance; HRD cancer	TROP2
NCT05489211	Biliary Tract Cancer; Endometrial Cancer; Gastric Cancer; Prostate Cancer; Ovarian Cancer; Urothelial Cancer	TROP2
NCT05327530	Urothelial Cancer	TROP2
NCT05456685	Fallopian Tube Cancer; Ovarian Cancer; Primary Peritoneal Cancer	FRα
NCT04538742	Breast Cancer	HER2
NCT05149768	Cutaneous Systemic Sclerosis	CD30
NCT03386513	Blastic Plasmacytoid Dendritic Cell Neoplasm; Myeloproliferative Neoplasm	CD123
NCT03288545	Urothelial Cancer; Renal Pelvis Neoplasms; Ureteral Neoplasms; Urethral Neoplasms; Urinary Bladder Neoplasms; Urologic Neoplasms	NECTIN-4
NCT06827236	Breast Cancer	HER2
NCT05579366	Breast Adenocarcinoma; Endometrial Cancer; Non-Small Cell Lung Cancer; Fallopian Tube Cancer	FRα
**Trial Phase 3**		
NCT04595565	Breast Cancer	HER2
NCT06161025	Solid Tumor	CDH6
NCT06203210	Small Cell Lung Cancer	B7-H3
NCT03734029	Breast Cancer	HER2
NCT05950945	Breast Cancer	HER2
NCT03523585	Breast Cancer	HER2
NCT03529110	Breast Cancer	HER2
NCT05754853	Urothelium Cancer	HER2
NCT03474107	Bladder Cancer; Ureteral Cancer; Urothelial Cancer	NECTIN-4
NCT05870748	Ovarian Cancer; Fallopian Tube Cancer; Primary Peritoneal Cancer	FRα
NCT06132958	Endometrial Cancer	TROP2
NCT01712490	Lymphoma	CD30
NCT05629585	Breast Cancer	TROP2
NCT05374512	Breast Cancer	TROP2
NCT06074588	Non-Small Cell Lung Cance	TROP2
NCT04494425	Breast Cancer	HER2
NCT05445778	Fallopian Tube Cancer; Ovarian Cancer; Peritoneal Cancer	FRα
NCT06112379	Breast Cancer	HER2
NCT05687266	Non-Small Cell Lung Cance	TROP2
NCT04639986	Breast Cancer	HER2
NCT05609968	Non-Small Cell Lung Cance	TROP2
NCT06619236	Ovarian Cancer	FRα
NCT06841354	Triple Negative Breast Neoplasms	TROP2
NCT05104866	Breast Cancer	TROP2
NCT06103864	Breast Cancer	TROP2
NCT06989112	Endometrial Cancer	HER2
**Trial Phase 4**		
NCT06429761	Breast Cancer	HER2

### Combination strategies: mechanistic synergy and clinical expansion

Despite the substantial clinical success of ADC monotherapy, challenges such as limited response rates, acquired resistance, and immune evasion have driven growing interest in combination treatment strategies [182–184]. By leveraging mechanistic complementarity, ADCs are increasingly being integrated with immunotherapy [185–187], targeted agents [188, 189], chemotherapy [190, 191], and cellular therapies [192–194] to maximize therapeutic efficacy and expand patient benefit. These combinatorial approaches aim to enhance tumor cell killing, overcome resistance mechanisms, and reshape the TME in favor of sustained antitumor immunity. One of the most actively explored strategies is the combination of ADCs with immune checkpoint inhibitors (ICIs) [127]. Mechanistically, several ADCs have been shown to induce ICD, characterized by the release of DAMPs, which promote dendritic cell activation, antigen cross-presentation, and subsequent priming of tumor-specific T cells [117]. Preclinical models and early-phase clinical trials support the hypothesis that ADC-induced ICD may sensitize tumors to checkpoint blockade [195–198]. For instance, T-DXd combined with durvalumab (an anti-PD-L1 ICI) is currently being evaluated in HER2-positive breast cancer and NSCLC, with preliminary findings suggesting potential clinical activity (DESTINY-Breast07, NCT04538742; DESTINY-Lung03, NCT04686305). Similarly, sacituzumab govitecan combined with atezolizumab (an anti-PD-L1 ICI) is under investigation in TNBC [199]. Beyond checkpoint inhibitors, alternative immuno-oncology combinations are also under investigation. Experimental strategies incorporating ADCs with innate immune stimulators, such as STING or TLR agonists, aim to convert immunologically “cold” tumors into “hot” ones and enhance responsiveness to downstream adaptive immune effectors [200–202].

Beyond immunotherapy, ADCs are increasingly being combined with molecularly targeted agents to disrupt compensatory survival pathways and overcome resistance mechanisms. For example, telisotuzumab vedotin, a c-MET-directed ADC, has been evaluated in combination with erlotinib, an EGFR tyrosine kinase inhibitor (TKI), in c-MET-positive and EGFR-mutant NSCLC [203, 204]. The Phase Ib/II study (NCT02099058) showed preliminary antitumor activity, supporting dual oncogenic pathway inhibition. Similarly, Nectin-4-targeted ADCs, such as enfortumab vedotin or experimental analogues, have been investigated in preclinical models of pancreatic and bladder cancer [205, 206]. These studies demonstrated that autophagy is induced as a pro-survival response to ADC-mediated cytotoxic stress, and that combining with autophagy inhibitors (e.g., chloroquine or LY294002) significantly enhances tumor cell apoptosis and delays tumor growth in xenograft models (e.g., HT1376 and BxPC-3) [207, 208]. Furthermore, DNA-damaging ADCs, such as topoisomerase I inhibitors conjugated to antibodies (e.g., sacituzumab govitecan), are being explored in combination with PARP inhibitors (e.g., Olaparib, niraparib, or talazoparib) in BRCA-mutant or homologous recombination-deficient (HRD) tumors to enhance synthetic lethality [209], and are also being investigated in TNBC, where DNA repair deficiencies are often present (NCT04039230). While clinical trials in this area are still limited, the rationale is supported by mechanistic synergy. However, overlapping hematologic toxicity, particularly neutropenia, and thrombocytopenia, remains a key safety concern that may limit the tolerability of this combination [210–213].

Conventional chemotherapy may also potentiate ADC activity by modifying the TME, increasing tumor permeability, or promoting antigen exposure [214, 215]. Sequential or concurrent administration strategies are being tested to determine optimal timing and dosing [44, 216]. For instance, a study on mirvetuximab soravtansine demonstrated that concurrent administration with paclitaxel in ovarian cancer models significantly enhanced antitumor activity compared to monotherapy [217]. Additionally, a recent clinical review of sacituzumab govitecan in breast cancer explored both sequential and concurrent administration with chemotherapy, emphasizing how different strategies affect toxicity profiles and therapeutic outcomes [218]. Moreover, novel approaches are emerging that combine ADCs with cell therapies or oncolytic viruses, with ADCs acting as immune-sensitizing agents to improve antigen release and immune priming before adoptive T-cell therapy [219, 220]. For example, a 2024 study by Taha et al. proposed a pharmacoviral platform where ADCs are used to sensitize tumors prior to treatment with VSVΔ51-based oncolytic viruses, effectively boosting T-cell recognition and activity against antigen-agnostic tumors [221]. Another 2025 report by Palma et al. emphasized the combinatorial potential of ADCs and oncolytic viruses in breast cancer, showing enhanced immune priming and T-cell infiltration when both are used in a timed regimen before adoptive cell transfer [222].

Looking ahead, the success of ADC-based combination therapies will hinge on rational trial design, proactive toxicity management, and biomarker-guided patient selection. As these strategies evolve, ADCs may shift from being single-agent cytotoxic tools to becoming pivotal elements within integrated, multimodal cancer treatment paradigms.

#### Current challenges and emerging research priorities

ADCs have achieved transformative success in cancer therapy over the past two decades, but significant obstacles remain in key domains, including target selection, structural design, drug resistance, and immune modulation. This section provides a comprehensive analysis of these ongoing challenges and highlights emerging directions in ADC research.

### Target selection: challenges and emerging innovations

The therapeutic efficacy of ADCs hinges on the feasibility and specificity of the target antigen [16, 122]. Most approved ADCs currently focus on targets such as HER2, CD30, CD33, and TROP2, which are characterized by high expression in malignant tissues, efficient internalization, and relevance to tumor biology [223–226]. However, ideal ADC targets remain scarce, especially in solid tumors, due to issues such as heterogeneous expression, low levels in normal tissues, or inadequate endocytic efficiency [100, 227]. Additionally, some tumors downregulate antigen expression during progression, leading to acquired resistance [228]. Therefore, current target selection strategies must evolve. Novel antigens associated with cancer stemness, immune escape, or metastasis, such as B7-H3, HER3, and CEACAM5, are being actively investigated [225, 229, 230]. Meanwhile, emerging techniques like spatial transcriptomics and single-cell multiomics are increasingly employed to improve the precision and translational potential of target discovery [230–232].

### Linker stability: challenges and structural innovations

As a molecular bridge between the antibody and payload, the linker plays a decisive role in determining both systemic stability and intracellular release kinetics [74, 233]. The instability of early-generation ADCs, such as gemtuzumab ozogamicin, highlighted the clinical risks of premature drug release [234]. Although current linkers like Val-Cit and disulfide-based structures have improved release specificity [235, 236], they still face challenges such as suboptimal plasma stability and unpredictable release timing [237]. To overcome these issues, two main strategies have emerged: (a) development of next-generation “smart” linkers that respond to multiple tumor-specific stimuli, such as pH, enzymatic activity, and redox conditions [238, 239]; and (b) co-optimization with antibody engineering technologies, including site-specific conjugation to reduce linker exposure and increase drug-release selectivity [240–242]. Additional innovations such as self-immolative linkers and PEGylation strategies also show promise in enhancing pharmacokinetic profiles and controlling payload behavior [243, 244].

### Drug resistance: mechanisms and strategic responses

Despite their molecular specificity and targeted delivery, ADCs are not immune to the development of therapeutic resistance, particularly under conditions of prolonged exposure or selective pressure [245]. Multiple resistance mechanisms have been identified that compromise intracellular drug accumulation and attenuate cytotoxic efficacy [29, 246]. Among these, downregulation or complete loss of the target antigen is one of the most frequently observed escape routes, which may occur through genetic alteration, epigenetic silencing, or antigen shedding. In addition, mutations that diminish antibody–antigen binding affinity, along with alterations in endocytic machinery or intracellular trafficking routes, can impair ADC internalization and lysosomal delivery. Lysosomal dysfunction, whether through altered pH, reduced enzymatic activity, or defective membrane transport, further limits payload activation and effective drug release. A notable consequence of sustained ADC exposure is the induction of multidrug resistance (MDR) phenotypes, particularly via the upregulation of ATP-binding cassette (ABC) efflux transporters such as P-glycoprotein. These transporters actively extrude the released cytotoxic agents, thereby lowering intracellular drug concentrations and accelerating therapeutic clearance [247, 248]. Moreover, tumor cells may acquire resistance to specific payload classes, such as microtubule inhibitors or topoisomerase I inhibitors, through enhanced DNA repair capacity, overexpression of anti-apoptotic proteins, or remodeling of cell cycle checkpoints that buffer against lethal damage [249]. In some cases, clathrin-mediated endocytosis itself may be downregulated, or lysosomal targeting disrupted, further obstructing the intracellular trafficking essential for ADC function [100].

To address these multifactorial barriers, a growing array of next-generation strategies is under development. One promising direction involves the design of dual-targeting ADCs that simultaneously engage two distinct tumor-associated antigens, thereby increasing target recognition redundancy and mitigating the impact of antigen heterogeneity or loss [36, 38, 250]. Incorporating membrane-permeable payloads with potent bystander effects enables ADCs to eliminate neighboring antigen-negative tumor cells, extending therapeutic reach within heterogeneous lesions [251, 252]. In parallel, the exploration of non-canonical targets, such as immune-modulatory molecules or metabolic antigens, aims to circumvent classical resistance pathways and reduce selective pressure on conventional tumor markers [253, 254]. Beyond antigen and payload design, researchers are engineering ADCs with immune-stimulatory components, such as toll-like receptor (TLR) or STING agonists, capable of triggering ICD and enhancing dendritic cell activation [109, 255]. These immunomodulatory ADCs may synergize with checkpoint blockade or other immunotherapies, bridging innate and adaptive anti-tumor responses. Emerging modalities, including proteolysis-targeting chimera (PROTAC)-ADCs, represent an innovative class of degradation-based therapeutics that selectively eliminate previously undruggable intracellular proteins, broadening the pharmacologic landscape of ADC technology [256, 257]. Moreover, rational combination strategies integrating ADCs with PARP inhibitors, ICIs, or standard chemotherapies are showing preclinical and clinical promise for enhancing efficacy and delaying resistance onset [258]. These approaches can be further potentiated by biomarker-driven adaptive treatment paradigms, wherein serial molecular profiling (e.g., liquid biopsy or functional imaging) informs real-time modulation of therapeutic regimens in response to evolving resistance dynamics [259–262]. Together, these advances are steering ADC development from static cytotoxic constructs to dynamic, programmable therapeutics capable of overcoming resistance and sustaining durable responses.

### Immune modulation: a barrier to ADC efficacy

Beyond intrinsic cellular resistance, the TME plays a pivotal role in modulating ADC efficacy. Many solid tumors are characterized by a profoundly immunosuppressive milieu, marked by the infiltration of regulatory T cells (Tregs), myeloid-derived suppressor cells (MDSCs), and tumor-associated macrophages (TAMs), which collectively dampen anti-tumor immune responses and promote immune escape [263–265]. Importantly, most approved ADCs employ payloads that exert direct cytotoxicity without triggering robust ICD, thereby limiting their capacity to stimulate adaptive immune responses [266]. In contrast, certain newer payloads, such as tubulysins, duocarmycins, or DNA-damaging agents, have been shown to promote ICD and enhance dendritic cell activation and T-cell priming in preclinical models [267, 268]. Still, the immunostimulatory potential of ADCs remains underutilized in clinical settings. Another major concern is that continuous ADC exposure may inadvertently remodel the immune landscape, promoting immune evasion or checkpoint upregulation in resistant tumors [266]. Furthermore, payload-induced inflammation can recruit immunosuppressive cells or trigger stromal remodeling that limits drug penetration [269].

#### Translational challenges from bench to bedside

ADCs, as a class of biotherapeutics that combine immunological targeting precision with potent cytotoxic payloads, have demonstrated transformative clinical breakthroughs across diverse malignancies, including breast cancer, lymphoma, and lung cancer [216, 270, 271]. Nevertheless, translating ADCs from preclinical research to routine clinical practice continues to face considerable obstacles across multiple technical and systemic dimensions [272, 273]. This section provides a comprehensive overview of the translational challenges associated with dose optimization, manufacturing control, clinical trial design, and commercialization, and proposes directions for overcoming these barriers.

### Translational barriers: laboratory discovery and clinical application

The transition of ADCs from laboratory prototypes to clinical candidates presents a distinct set of challenges stemming from their structural complexity and multimodal mechanisms of action [16]. Unlike conventional therapeutics, ADCs must be optimized not only for pharmacological activity but also for manufacturability, stability, and patient selectivity [122, 274, 275]. Broadly, the translational bottlenecks can be summarized into three core categories. Initially, dose optimization remains inherently difficult due to the narrow therapeutic window of ADCs, where efficacy must be carefully balanced against off-target toxicity [276–278]. For example, T-DXd has been associated with ILD due to its topoisomerase I inhibitor payload [279], while SN-38-containing ADCs often lead to dose-limiting myelosuppression [58, 280]. Additionally, the pharmacokinetics of payload release is often unpredictable, with unstable linkers such as Val-Cit prone to premature cleavage in plasma [72]. Moreover, the “bystander effect”, while beneficial in targeting heterogeneous tumors, also raises the risk of collateral toxicity in normal tissues [281, 282]. To address these issues, dosing strategies guided by pharmacokinetic modeling (e.g., the 5.4 mg/kg every three weeks regimen for T-DXd) and patient stratification based on antigen levels (e.g., HER2-low) or permeability-related biomarkers (e.g., TROP2 mRNA) have been explored [43, 283, 284].

In addition, manufacturing complexity poses a major barrier to scalability. ADCs are composed of three distinct components, monoclonal antibody, linker, and cytotoxic drug, each contributing to variability in quality attributes [285, 286]. Traditional random conjugation methods result in heterogeneous DAR, as seen with early-generation gemtuzumab ozogamicin (DAR range 2-6) [20, 62]. Moreover, hydrophobic payloads like MMAE tend to induce aggregation, compromising stability [78, 287]. To improve consistency, site-specific conjugation platforms such as engineered cysteine residues (e.g., Thiomab) or enzymatic ligation have been introduced [288–290], enabling fixed DAR values (e.g., DAR = 8 in T-DXd). The incorporation of hydrophilic linkers, such as CL2A in Trodelvy, has also improved plasma stability and half-life, facilitating industrial-scale production [291, 292]. Furthermore, clinical trial design must contend with target expression heterogeneity and evolving resistance mechanisms [293]. For instance, variable expression of TROP2 in TNBC has been linked to reduced response rates, with ASCENT reporting an ORR of only 35% [135, 294]. Resistance may also develop via antigen downregulation, impaired endocytosis, or lysosomal dysfunction [295]. In response, combinatorial approaches are being pursued [296, 297]. ADC-induced ICD offers a rationale for synergistic combinations with ICIs, as demonstrated by trials combining T-DXd with durvalumab in HER2-positive NSCLC (NCT04538742) [298, 299]. Furthermore, innovative basket trial designs (e.g., DESTINY-PanTumor02: NCT04494425) are being employed to evaluate efficacy across tumor types, expanding the potential clinical utility of ADCs [110].

### Diagnostic precision: target assessment and patient stratification

The clinical success of ADCs is closely tied to accurate patient stratification, particularly in cancers characterized by high heterogeneity and dynamic antigen expression [46]. The integration of CDx into early-phase development is essential for maximizing therapeutic precision and safety [300–302]. However, the current landscape reveals multiple limitations in diagnostic tools, which hinder optimal patient selection and broader adoption of ADCs [85, 303]. Commonly used target detection methods such as immunohistochemistry (IHC) and fluorescence in situ hybridization (FISH) are subject to inter-laboratory variability, ambiguous cutoff definitions, and limited quantitative precision [304, 305]. For instance, although standardized IHC-based assessment of TROP2 is commonly used in ADC clinical trials, variability in antibody clones, staining procedures, and scoring methods can introduce interpretive inconsistencies. These factors, while not systematically quantified, are widely acknowledged to influence biomarker-based enrollment decisions. Moreover, many ADC programs still rely on single-threshold biomarker definitions (e.g., IHC 2 +/3 +) that fail to account for temporal and spatial variation in target expression, TME context, or drug penetration potential [259, 306, 307].

Even in approved ADC targets such as HER2 and CD30, expression variability across tissues and histological subtypes has been observed [308, 309]. The success of T-DXd in HER2-low breast cancer highlights the existence of responsive subpopulations previously considered antigen-negative, underscoring the need for more sensitive and discriminating diagnostic platforms [110, 310, 311]. Similar challenges are noted in low CD30-expressing T-cell lymphomas and TROP2 expression patterns in lung cancer [312, 313]. Emerging solutions are advancing on several fronts. Radiolabeled antibodies (e.g., 89Zr-DFO-trastuzumab) combined with PET imaging offer whole-body spatial resolution of antigen distribution, overcoming the sampling limitations of tissue-based assays [314, 315]. Meanwhile, multi-omics technologies, such as spatial transcriptomics and single-cell proteomics, provide nuanced insights into antigen-microenvironment interplay and may inform predictive modeling of ADC response [317]. The co-development of CDx products alongside ADCs, ensuring alignment with indications, clinical workflows, and regulatory expectations, is increasingly recognized as a cornerstone for future precision deployment of ADC therapies [318, 319].

### Commercial prospects: cost challenges and clinical translation

Despite impressive clinical outcomes, the commercialization of ADCs remains encumbered by high production costs, pricing pressure, and the need to establish clear value propositions for reimbursement [320, 321]. These economic and regulatory complexities are compounded by the unique structural and pharmacological challenges inherent to ADCs, making sustainable market access a formidable task [322, 323]. Manufacturing costs are significantly higher for ADCs than for conventional mAbs, largely due to the complexity of integrating toxic payloads, linkers, and conjugation technologies into a tightly regulated workflow [324]. Synthesis of certain payloads, such as PBD dimers, may require more than ten high-purity synthetic steps. Further, aseptic conjugation processes and stringent impurity controls raise both technical barriers and operational costs [325]. To mitigate these challenges, the field is moving toward platform-based manufacturing approaches, such as Synaffix's GlycoConnect, and adopting continuous-flow microreactor systems that achieve yields exceeding 90%, thereby improving scalability and cost-efficiency [326]. Pricing pressure is equally critical. As highlighted in a recent IQVIA white paper on novel oncology therapies, many ADC regimens have treatment costs exceeding $100,000 per course, making reimbursement increasingly dependent on demonstrated clinical benefit and cost-effectiveness [327]. For example, T-DXd extended median OS from 16.8 to 23.4 months in HER2-low breast cancer [110], while Trodelvy reduced subsequent chemotherapy need by 52% in TNBC [135, 328]. Real-world evidence (RWE) is also being integrated into health technology assessments to better define the value proposition of ADCs in varied healthcare settings [328–330].

Looking forward, the commercial landscape for ADCs is expanding. Next-generation ADCs with dual targeting capabilities (e.g., ZW49 targeting HER2/EGFR) address tumor heterogeneity and resistance [16, 331, 332]. Meanwhile, non-cytotoxic payloads, such as PROTAC-ADCs, aim to unlock previously undruggable targets [266, 333]. Diagnostic innovation is also accelerating: PET imaging using radiolabeled antibodies (e.g., 89Zr-trastuzumab) is emerging as a high-resolution alternative to conventional IHC [314]. Recent market analyses project that the global ADCs market will exceed $16.5 billion by 2030, with a compound annual growth rate (CAGR) surpassing 10% [334]. This growth is primarily driven by the expanding application of ADCs in earlier lines of therapy and neoadjuvant settings, as well as the advancement of novel targets such as B7-H3 and DLL3 in refractory cancers [313, 335, 336]. Additionally, increasing demand for contract manufacturing services reflects rapid industry scale-up and technological progress, facilitating the clinical translation and commercialization of next-generation ADC therapies. The successful clinical translation of ADCs depends not only on cutting-edge molecular design but also on a cohesive integration of development, manufacturing, regulatory, and reimbursement strategies. Bridging the translational gap requires precise dosing, scalable and consistent production, innovative trial designs, and sustainable pricing models [320, 337]. As bispecific ADCs, intelligent linkers, and functional payloads continue to evolve, ADCs are poised to transition from costly breakthrough therapies to foundational pillars of targeted cancer care [338–340]. Strategic foresight in global supply chain coordination and combination therapy ecosystems will be key to realizing this transformation.

#### Emerging technologies and potential applications

**Fig. 6:**
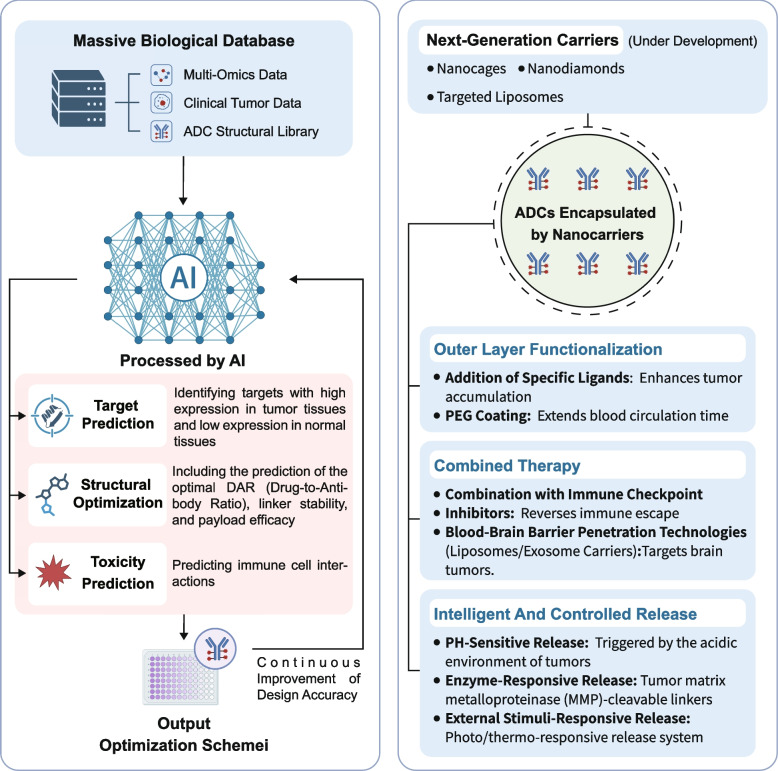
Emerging strategies for enhancing ADC precision and delivery. Two major avenues are being explored to optimize ADC efficacy. On the left, AI-based platforms integrate multi-omics data, clinical tumor profiles, and structural libraries to guide target identification, structural refinement (e.g., DAR, linker type), and toxicity prediction. This facilitates iterative optimization of ADC design. On the right, nanocarrier-based systems, including nanocages, nanodiamonds, and targeted liposomes, are under development to improve tumor accumulation, prolong circulation, and support controlled drug release. Functionalization strategies, such as ligand addition and PEGylation, along with intelligent release mechanisms triggered by pH, enzymatic activity, or external stimuli, offer promising avenues to enhance therapeutic precision and bioavailability.

As the field of ADCs continues to evolve, several emerging technologies are shaping the future of ADC development. These innovations have the potential to address current limitations, enhance the therapeutic efficacy of ADCs, and push the boundaries of cancer treatment [341–343]. In this section, we explore some of these key technological advances, including artificial intelligence (AI)-driven ADC design, the role of nanotechnology in drug delivery, and the development of dual-targeting strategies and personalized treatments (Fig. 6). Meanwhile, we also briefly discuss antibody-guided delivery platforms that go beyond the classical linker-payload paradigm, including immunoliposomes and engineered exosomes, which may offer complementary strategies for tumor targeting.

### AI-driven ADC design: target selection and therapeutic efficacy

AI has rapidly become a powerful tool in drug development, including the design of ADCs [39, 344, 345]. In the past, the process of selecting suitable targets for ADCs often relied heavily on empirical data and labor-intensive experimentation [346, 347]. However, the integration of AI into the ADC design process allows for a more data-driven approach, which can significantly speed up the discovery phase and improve the precision of the design [348–350]. AI-based algorithms can analyze vast amounts of biological data, identifying potential tumor-associated antigens (TAAs) with high expression in tumor cells and minimal expression in normal tissues [351–353]. This enables more accurate target identification, improving the selectivity of ADCs and reducing the risk of off-target toxicity [354]. Additionally, AI can be used to predict the optimal DAR [355], linker stability [356], and payload selection [357] by simulating the interactions between the antibody, linker, and drug payload in silico [358]. This approach can lead to better-controlled ADC formulations, enhancing therapeutic outcomes [359, 360].

Furthermore, AI can assist in predicting the toxicity profiles of ADCs, including the potential for off-target effects [361, 362]. By modeling how ADCs interact with various biological systems, AI algorithms can help design ADCs that minimize toxicity while maximizing therapeutic efficacy [363–365]. Recent studies have demonstrated that AI can also predict the interaction of ADCs with immune cells, thus optimizing their immunogenicity and enhancing the immune response against tumors [366, 367]. This enables the development of more effective ADC therapies, especially for cancers with immune evasion mechanisms [368, 369]. As AI technologies evolve, the ability to model complex biological systems in real-time will further improve the precision and adaptability of ADC designs, allowing for continuous refinement during the development process [370, 371]. These advancements will ultimately lead to more personalized and targeted cancer treatments [371, 372]. AI-driven approaches could also help in overcoming current limitations in ADC therapies, such as drug resistance and poor tumor penetration, by optimizing the design and formulation of ADCs tailored to specific TME [373, 374]. As AI technologies continue to advance, their integration into ADC development will likely result in faster and more efficient design cycles, improving the overall success rate of ADC-based therapies [375, 376].

### Nanotechnology: drug delivery and targeting capabilities

Nanotechnology is emerging as a crucial tool in enhancing the drug delivery capabilities of ADCs, particularly in addressing the challenges of tumor targeting and drug penetration [377–379]. Traditional chemotherapy often suffers from inefficient drug delivery, leading to suboptimal efficacy and significant side effects due to the inability of the drugs to selectively accumulate in tumor tissues [380–382]. Nanotechnology offers a promising solution by providing innovative drug delivery systems that can improve the pharmacokinetics of ADCs and enhance their tumor-targeting abilities [383–385]. Nanocarriers, such as lipid nanoparticles (LNPs), polymeric nanoparticles, and micelles, have emerged as vehicles that can encapsulate ADCs, protecting them from premature degradation in circulation while enhancing their stability and bioavailability [386–388]. These carriers can be specifically engineered to improve the accumulation of ADCs at the tumor site by modifying their surface properties [389, 390]. One innovative approach includes the incorporation of targeting ligands, such as peptides, antibodies, or aptamers, that bind to tumor-specific receptors, increasing the likelihood of ADCs accumulating in tumor tissues [391–394]. For example, targeting overexpressed receptors like HER2, folate receptors, and integrins in certain tumors has been shown to enhance the selectivity of nanocarriers [395–397].

Moreover, nanotechnology presents an innovative solution to delivering ADCs to difficult-to-reach tumors, such as brain tumors [398–400]. The blood-brain barrier (BBB) is a major obstacle to drug delivery in the central nervous system (CNS), making it extremely difficult to treat brain cancers with traditional chemotherapy [401, 402]. Nanocarriers, however, have shown promising results in crossing the BBB and delivering ADCs directly to brain tumors [403–405]. Recent research has demonstrated the potential of using liposomes and exosome-based systems for targeted drug delivery across the BBB, providing hope for treating glioblastomas and other CNS cancers that are otherwise resistant to conventional therapies [406–409]. In addition to enhancing targeting, nanotechnology enables controlled release mechanisms that improve therapeutic efficacy [410]. pH-sensitive nanoparticles, for instance, can release their cytotoxic payload selectively in the acidic microenvironment of tumors [411, 412]. This feature allows for higher concentrations of the drug to accumulate within the tumor, while minimizing exposure to surrounding healthy tissues [413, 414]. Such "smart" nanocarriers are equipped with stimuli-responsive properties, such as enzymatic cleavage or temperature sensitivity, which ensure that the ADCs are released only at the tumor site or under specific conditions, maximizing efficacy and reducing systemic toxicity [415–417]. The clinical translation of nanotechnology in ADC delivery is progressing rapidly, with numerous preclinical studies and a few early-phase clinical investigations underway [418, 419]. The use of lipid-based nanoparticles for delivering chemotherapeutic agents, such as Doxil (PEGylated liposomal doxorubicin), has already demonstrated improved drug bioavailability and reduced systemic toxicity [420, 421]. Inspired by such success, researchers have begun developing nanoformulations of ADCs, including PEGylated and lipid-based nanoparticle systems, to enhance pharmacokinetics, tumor accumulation, and therapeutic index [388]. While most of these nano-ADC platforms are still in the preclinical stage, some are progressing toward clinical evaluation for improved tumor targeting and reduced off-target effects [422–425]. One notable example is the Phase I clinical study of anetumab ravtansine, a mesothelin-targeting ADC conjugated with the maytansinoid DM4, which was investigated in combination with pegylated liposomal doxorubicin (PLD) in patients with platinum-resistant ovarian, fallopian tube, or primary peritoneal cancer [426, 427]. This combination leverages both ADC specificity and nanoparticle-based drug delivery to enhance antitumor activity and limit toxicity (NCT02638926). Although the ADC itself was not encapsulated in nanoparticles, this trial reflects a broader strategy of integrating nanotechnology principles with ADC therapy to optimize efficacy and safety.

Furthermore, the combination of nanocarriers with ICIs represents a promising approach to enhance the therapeutic efficacy of ADCs [428, 429]. Nanoparticles, such as lipid-based carriers, can be used to co-deliver ICIs alongside ADCs, helping to overcome immune evasion mechanisms in tumors [430–432]. For example, nano-immunoconjugates that combine ADCs with ICIs have shown potential in preclinical models, demonstrating synergistic effects and improved anti-tumor responses [433, 434]. These therapies are currently undergoing early-phase clinical trials and have shown promise in cancers traditionally difficult to treat with ADCs alone [435]. Notable trials include the combination of Enfortumab vedotin with ICIs in urothelial cancer, which has shown significant improvements in OS and PFS compared to traditional chemotherapy [205, 436, 437]. Additionally, clinical studies on SGN-PDL1V, a mesothelin-targeting ADC conjugated with vedotin, have demonstrated its potential when used in combination with ICIs, with early-phase data showing a favorable response (NCT05208762) [438]. These early results indicate that combining ADCs with ICIs via nanocarriers could be a game-changing strategy in overcoming tumor resistance mechanisms and improving patient outcomes [439, 440]. Looking forward, the development of next-generation nanocarriers, such as nanocages [441], nanodiamonds [442], and targeted liposomes [443], could significantly enhance the precision of ADC therapies [444]. These advanced carriers could offer higher payload delivery, more accurate targeting, and better controlled-release systems [445–447]. Additionally, the integration of AI and machine learning into the design of these nanocarriers promises to accelerate the optimization of ADC formulations, enabling highly personalized and effective cancer treatments in the future [448–450].

### Dual-targeting and personalized treatment

Dual-targeting refers to a therapeutic strategy where ADCs are designed to simultaneously bind to two different tumor antigens on the surface of cancer cells [451–453]. This approach addresses challenges like tumor antigen heterogeneity and resistance mechanisms by targeting multiple sites on tumor cells, which can enhance therapeutic efficacy [454]. By combining two targeting domains, dual-targeting ADCs increase the precision of treatment, particularly in tumors with varying levels of antigen expression [455, 456]. This strategy has emerged as a promising way to overcome some limitations of conventional single-target ADCs, improving targeting accuracy and tumor cell killing in difficult-to-treat cancers [457]. For example, bispecific ADCs that target both HER2 and EGFR have shown promising results in preclinical models by increasing the breadth of tumor targeting [458]. Additionally, MM-111, a bispecific antibody targeting both HER2 and HER3, has demonstrated its potential in HER2-positive breast cancer, especially in overcoming resistance mechanisms driven by HER3 activation (NCT01097460) [459]. By engaging two different tumor-associated antigens, these dual-targeting ADCs may be more effective at overcoming tumor heterogeneity and preventing immune escape [460–462]. Similarly, TJ101, which targets EGFR and B7-H3, has shown promising results in NSCLC, HNSCC, and colon cancer. These bispecific ADCs are significantly superior to monoclonal anti-EGFR or anti-B7-H3 ADCs and have a lower toxicity profile [463]. Alongside dual-targeting strategies, personalized treatment is becoming an integral part of cancer therapy, and ADCs are no exception [464, 465]. The advent of precision medicine has allowed for the development of ADCs that are tailored to the genetic and molecular characteristics of individual patients' tumors [466–468]. By analyzing the specific genetic mutations, biomarker expression, and molecular profiles of a patient's cancer, ADCs can be designed to target the unique features of the tumor [469, 470]. This approach enhances treatment efficacy and minimizes the risk of adverse effects by ensuring that the therapy is specifically targeted to the tumor cells [471]. Moreover, new technologies such as multi-omics integration, CRISPR gene editing, and liquid biopsy are further advancing personalized ADC design [472–474]. Multi-omics allows for a deeper understanding of the tumor's genetic and protein profile, ensuring ADCs target key molecular features [475]. CRISPR enables the creation of patient-specific tumor models to test ADC efficacy [476], while liquid biopsy provides real-time tumor information, allowing for adaptive treatment strategies [477]. These technologies collectively ensure that ADCs are better tailored to individual tumors, improving efficacy and reducing side effects [478, 479].

In addition to these advancements in ADC design, the use of CDx is becoming increasingly important to identify patients most likely to benefit from these therapies [480, 481]. CDx involves tests and tools that help match the right treatment to the right patient based on the genetic, molecular, or protein characteristics of their tumor [482–484]. For example, HER2 testing has been critical in determining which patients are suitable for HER2-targeted ADCs like trastuzumab emtansine (Kadcyla®), which is used in HER2-positive breast cancer [485, 486]. This test ensures that only patients with high HER2 expression, who are more likely to respond to the therapy, receive the treatment. Similarly, PD-L1 expression testing is an essential tool for selecting patients who would benefit from ADC therapies that combine ICIs with cytotoxic drugs, such as atezolizumab (Tecentriq®) combined with cytotoxic payload MMAE [487, 488]. As more ADCs targeting a variety of new biomarkers are developed, such as CD66c, SSEA-4, and CDH17, the role of CDx will continue to grow, further personalizing treatment [489–491]. For instance, the ADC targets CD66c, a glycosylated protein overexpressed in gastroesophageal junction cancer [489]. Companion diagnostic tests can identify patients with high CD66c expression, ensuring that only those who are likely to respond to this targeted therapy receive the treatment, thus improving therapeutic outcomes and minimizing unnecessary side effects [492]. In the future, liquid biopsy and multi-omics technologies will likely further refine these diagnostic tools, allowing clinicians to monitor tumor evolution in real-time and adapt treatment strategies accordingly [493–495]. These advancements will ensure that the right therapy is matched to the right patient, optimizing therapeutic outcomes while minimizing unnecessary side effects, thus significantly improving the precision and success of ADC therapies [154, 496, 497].

**Fig. 7:**
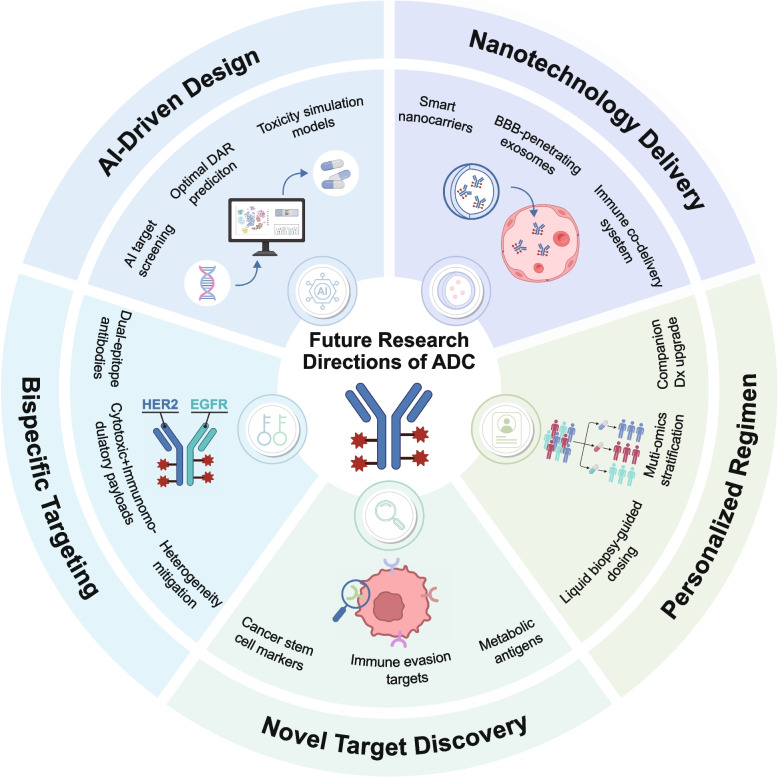
Future research directions in ADC development. Five major themes are anticipated to shape the future of ADC research. (1) AI-driven design will refine DAR optimization, target selection, and toxicity prediction through simulation models and high-throughput data integration. (2) Nanotechnology-based delivery, including smart carriers and blood–brain barrier-penetrating exosomes, is expected to enhance tumor penetration and facilitate immune co-delivery. (3) Personalized regimens, supported by multi-omics stratification, CDx, and liquid biopsy-guided dosing, will enable individualized therapy. (4) Novel target discovery is focusing on cancer stem cell markers, immune evasion molecules, and metabolic antigens. (5) Bispecific targeting strategies, which employ dual-epitope or bispecific antibodies and combine cytotoxic with immunomodulatory payloads, aim to overcome tumor heterogeneity and therapeutic resistance.

As ADC technology continues to progress, the integration of emerging technologies such as AI, nanotechnology, and dual-targeting strategies will undoubtedly shape the future of cancer therapy [498–500]. AI-driven design will enhance the speed and precision of ADC development, while nanotechnology will improve drug delivery and targeting, particularly in difficult-to-treat tumors. Dual-targeting ADCs, combined with personalized treatment approaches, hold the potential to overcome challenges like antigen heterogeneity and resistance, offering new hope for patients with complex cancers. Together, these innovations promise to revolutionize the ADC landscape, bringing us closer to achieving more effective, safer, and individualized cancer therapies [501, 502] (Fig. 7).

### Other non-linker antibody–drug delivery platforms

Beyond the classical antibody-linker-payload architecture, non-linker or carrier-based platforms utilize an antibody or ligand to guide drug-loaded carriers, rather than chemically conjugating the payload via a cleavable linker [503, 504]. Notably, carrier systems such as immunoliposomes, antibody-functionalized polymer or lipid nanoparticles, protein-based nanocages, and engineered exosomes have increasingly been investigated as modular alternatives to conventional ADCs [425, 505, 506]. A representative example is MM-302, a HER2-targeted liposomal formulation of doxorubicin incorporating a surface-displayed anti-HER2 scFv fragment for tumor targeting [507]. Early-phase studies demonstrated targeted tumor accumulation and reduced cardiotoxicity compared to free doxorubicin, suggesting its potential as a safer HER2-targeted delivery system [508, 509]. Engineered exosomes represent another branch of carrier-based strategies, which are cell-derived extracellular vesicles capable of encapsulating chemotherapeutic agents or nucleic acid payloads and can be modified with antibodies or ligands for selective tumor targeting. Fc-binding extracellular vesicles (Fc-EVs) capture the Fc domain of antibodies, enabling modular assembly of targeting moieties such as anti-HER2 or anti-PD-L1 [503, 510]. When loaded with doxorubicin, these Fc-EVs demonstrated targeted tumor suppression and prolonged survival in melanoma models. These platforms theoretically support multi-antigen targeting and co-delivery of multiple payloads, thereby addressing key resistance mechanisms such as antigen heterogeneity, antigen loss, and pathway redundancy [511–514]. However, despite promising preclinical data, their clinical translation remains limited by challenges in manufacturing reproducibility, pharmacokinetic predictability, and regulatory validation [515, 516]. For example, the phase II HERMIONE trial of MM-302 was discontinued following a futility analysis, as it failed to show a PFS benefit over chemotherapy plus trastuzumab (NCT02213744) [517]. These platforms represent a complementary approach to tumor-targeted therapy, especially in scenarios where classical ADCs are constrained by single-antigen dependency or payload limitations. Although still in early stages of development, such approaches may eventually enable multi-antigen targeting and combinatorial payload delivery, offering added flexibility in overcoming tumor heterogeneity and resistance.

#### Conclusions and perspective

ADCs, often described as biological “guided missiles,” have undergone rapid evolution in recent years, establishing themselves as a key therapeutic modality for both solid tumors and hematologic malignancies [518, 519]. As clinical applications broaden, however, ADCs are increasingly challenged by complexities in molecular engineering, target antigen selection, resistance mechanisms, combination strategies, toxicity profiles, and equitable global access [520–522]. Gaining mechanistic insight into these multifaceted bottlenecks is essential for informing the next generation of ADC design and optimizing their clinical integration across diverse oncologic settings.

### Balancing innovation with accessibility in ADC development

The evolution from early ADCs to third-generation constructs, such as T-DXd and sacituzumab govitecan, has brought improved DAR (e.g., fixed DAR = 8), selective release via cleavable linkers, and potent bystander effects. These features have significantly broadened clinical applications [523–525]. However, they also introduce new manufacturing complexity and cost burdens [526]. For example, site-specific conjugation and hydrophilic linker incorporation improve stability but require advanced GMP workflows and stringent quality control—systems often underdeveloped in low- and middle-income countries (LMICs) [527–530]. Geographic disparities are becoming increasingly evident: while Europe rapidly integrates ADCs targeting HER2-low and TROP2 into guidelines, many regions across Africa and Southeast Asia lack access due to diagnostic, logistic, and reimbursement barriers [266, 531–533]. This “access gap” suggests that future ADC innovation must balance scientific sophistication with manufacturability and affordability [534–536]. Platform-based, standardized production will be essential for broader global dissemination [537–540].

### From antigen dependency to mechanistic diversity in ADC design

The success of ADCs depends on ideal targets with high expression, low background, and efficient internalization, criteria fulfilled by HER2, CD33, TROP2, among others [541–544]. Yet, in real-world settings, antigen downregulation, heterogeneity, or internalization defects are common [545–547]. In HER2-positive breast cancer, for instance, 30–40% of patients develop reduced HER2 expression or altered trafficking after T-DM1 or T-DXd exposure, leading to diminished efficacy [548–552]. Moreover, ADC-induced cellular stress can trigger upregulation of ABC transporters (e.g., ABCB1, ABCG2), promoting drug efflux and resistance [553]. This forms a closed-loop of “targeting-response-escape” that undermines long-term benefit. Several strategies are emerging to address this [554, 555]. Dual-targeting ADCs (e.g., HER2/EGFR) increase antigen recognition redundancy [553, 556,557]; payloads such as TLR7/8 or STING agonists add immune-activating properties [558−560]; PROTAC-conjugated ADCs enable degradation of intracellular proteins beyond conventional druggable targets [561−563]. Collectively, these innovations represent a transition from “antigen dependency” to “mechanistic diversity” in ADC design [564, 565].

### Mechanism-based combination strategies for enhancing ADC efficacy

Although many ADCs exhibit superior monotherapy efficacy compared with conventional chemotherapy, their long-term therapeutic durability is frequently constrained by the emergence of resistance mechanisms, immune evasion, and dose-limiting toxicities [566−571]. In response, rationally designed combination regimens are being actively pursued to enhance clinical outcomes and overcome intrinsic limitations of ADC monotherapy. Notably, combinations of ADCs with immune checkpoint inhibitors have demonstrated early promise, supported by the mechanistic rationale that ADC-induced ICD may potentiate antitumor immune responses. For instance, T-DXd combined with durvalumab in the DESTINY-Breast07 trial, as well as sacituzumab govitecan in combination with atezolizumab in NSCLC and TNBC, are under clinical evaluation to assess the synergistic potential of this approach (NCT05382286) [572−576]. In parallel, preclinical studies have shown that Nectin-4-directed ADCs can be sensitized by autophagy inhibition, with agents such as chloroquine enhancing apoptotic cell death in pancreatic and bladder cancer models [577−580]. Furthermore, ADCs bearing the topoisomerase I inhibitor SN-38 have demonstrated synthetic lethality when combined with PARP inhibitors, particularly in tumors harboring BRCA mutations or HRD. However, overlapping hematologic toxicities remain a significant clinical consideration in these regimens, underscoring the importance of biomarker-guided dosing strategies and toxicity mitigation frameworks [581−584]. Importantly, combination strategies should follow a “mechanistic complementarity” principle, not simple drug stacking [585−587]. Biomarker-guided sequencing, real-time toxicity monitoring, and adaptive dosing will be essential to optimize synergy while minimizing harm [588,589].

### Innovative stratification tools for next-generation ADCs

Current patient stratification largely relies on IHC or FISH to assess target expression. However, this binary approach is increasingly inadequate in the context of antigen gradients and spatial heterogeneity, as exemplified by the clinical success of HER2-low ADCs [590−592]. Similar challenges are emerging with novel targets like TROP2 and B7-H3 [593−597]. Advanced diagnostic technologies, including PET-based immunotracers [598], spatial transcriptomics [599, 600], and liquid biopsies [601, 602], enable real-time profiling of target dynamics and TME evolution. These tools support a paradigm shift from “fixed-dose, fixed-interval” regimens to “feedback-driven, personalized” ADC administration, integrating antigen levels, drug penetration, and immune context [603, 604]. Collectively, ADC development stands at a pivotal inflection point, transitioning from single-function cytotoxic carriers to multidimensional therapeutic platforms. Future progress will depend not only on structural innovation but also on intelligent therapeutic design, rational combinations, and equitable implementation strategies. To fully unlock the potential of ADCs, it is essential to overcome the dual bottlenecks of molecular precision and real-world practicality. This entails designing agents that not only hit the target but also adapt to evolving biology and diverse clinical settings. While this review summarizes current trends and innovations, many of the discussed approaches remain exploratory, and further clinical validation will be required to confirm their long-term utility. Only through such integration can ADCs move beyond high-cost innovation and become foundational components of next-generation cancer therapy—across histologies, across geographies, and across healthcare systems.

## Data Availability

No datasets were generated or analysed during the current study.

## Abbreviations

ABC: ATP-binding cassette
ADCs: Antibody-drug conjugates
ADCC: Antibody-dependent cellular cytotoxicity
AML: Acute myeloid leukemia
BBB: Blood-brain barrier
CDx: Companion diagnostics
CNS: Central nervous system
DARs: Drug-to-antibody ratios
DAMPs: Damage-associated molecular patterns
DLBCL: Diffuse large B-cell lymphoma
FDA: Food and Drug Administration
FISH: Fluorescence in situ hybridization
HPB: Hepatopancreatobiliary
ICD: Immunogenic cell death
IHC: Immunohistochemistry
ILD: Interstitial lung disease
LNPs: Lipid nanoparticles
MDR: Multidrug resistance
MMAF: Monomethyl auristatin F
MMAE: Monomethyl auristatin E
NSCLC: Non-small cell lung cancer
OS: Overall survival
ORR: Objective response rate
PBD: Pyrrolobenzodiazepine
PEG: Polyethylene glycol
PFS: Progression-free survival
PLD: Pegylated liposomal doxorubicin
ROS: Reactive oxygen species
RWE: Real-world evidence
TAAs: Tumor-associated antigens
TAMs: Tumor-associated macrophages
TME: Tumor microenvironment
TNBC: Triple-negative breast cancer
TKI: Tyrosine kinase inhibitor
